# Psychosocial risk factors at work: perspectives of master’s and doctoral students working in the health sector*

**DOI:** 10.1590/1980-220X-REEUSP-2025-0045en

**Published:** 2025-07-07

**Authors:** Carolina Cassiano, Laura Andrian Leal, Elisabete Maria das Neves Borges, José Carlos Marques de Carvalho, Silvia Helena Henriques

**Affiliations:** 1 Universidade de São Paulo, Escola de Enfermagem de Ribeirão Preto, Ribeirão Preto, SP, Brazil.; 2 Universidade de São Paulo, Escola de Enfermagem de Ribeirão Preto, Departamento de Enfermagem Geral e Especializada, Ribeirão Preto, SP, Brazil.; 3 Escola Superior de Enfermagem do Porto, Porto, Portugal.

**Keywords:** Occupational Risks, Occupational Health, Health Personnel, Students, Health Postgraduate Programs

## Abstract

**Objective:**

To analyze psychosocial risk factors related to the work of graduate students in health.

**Method:**

An exploratory qualitative study, with master’s and doctoral students working in the health area, linked to five graduate programs of a Brazilian public higher education institution. Data collection took place between October and December 2023, using a sociodemographic, academic and professional questionnaire, and focus groups guided by a script. Deductive thematic analysis was used to interpret the data.

**Results:**

Sixty-two graduate students who worked participated in the study. The following psychosocial risk factors were identified in the work context: organizational culture and function; decision-making and control; organizational changes; worker role in organization; career development; interface between work and family; interpersonal relationships; harassment and violence. In work content, factors were setting and work equipment, task planning, work workload and pace, and schedule.

**Conclusion:**

Training centers and work organizations should pay attention to the psychosocial risk factors to which their graduate students who work are exposed so that strategies for their management can be incorporated.

## INTRODUCTION

Work in healthcare organizations is built through the articulation of distinct processes, in which each professional contributes with their own knowledge and tools of practice. In Brazil, there is primary care, which offers basic, preventive care and management of chronic conditions, and is the gateway to the Brazilian Health System, focused on continuous monitoring. Secondary care involves specialized services, such as consultations with experts and some more complex exams. Tertiary care is focused on advanced treatments, such as highly complex surgeries and intensive care. It is worth noting that each level of care aims to ensure adequate care according to patients’ needs^(1)^.

Such care modalities, as well as healthcare organizations, increasingly rely on integrated research teams to optimize the delivery of high-quality, evidence-based care that improves care experience^(2)^. Therefore, health workers must be trained to meet people’s daily health needs. This fact extends to *stricto sensu* graduate studies, and there is an increase in the number of workers applying for these programs, with a view to qualifying and developing research^(3)^.

In this way, there is a contribution to scientific development in health, which can positively impact care. Furthermore, healthcare is complex and diverse, and each profession in this area has unique characteristics that are necessary for complementary care. However, work, specifically in the health area, despite being a context conducive to the formation of identity, psychic meanings and social interactions, can also result in risks and illness^(4)^.

From this perspective, psychosocial risks at work refer to failures in the organization and management of the work context or content, which may cause psychological, physical, and social harm to workers^(5#x2013;7)^. These risks can be triggered by psychosocial risk factors, including work overload, lack of clarity in job responsibilities, low participation in decision-making, poor change management, job insecurity, ineffective communication, lack of support, harassment, and interactions with difficult individuals, as highlighted by the theoretical framework adopted in this study^(5,6)^.

The growing attention to psychosocial factors, risks and their potential consequences for workers’ health has gained prominence due to changes in the world of work. Recent changes in this global scenario have intensified existing psychosocial risk factors and introduced new ones to be considered. This promotes the opening of debates, both at national and international levels, on the need to prioritize this issue in policies, strategies and actions related to work^(8)^.

Linking this issue to the graduate population, researchers from the United Kingdom show that many doctoral students experience disappointment and stress during their studies, often related to a lack of support and the challenges of completing the course while working^(9)^.

Although graduate courses in health are essential for knowledge production, in meeting health demands, in addition to enabling professional qualification in the workplace, there is a scientific gap regarding psychosocial risk factors at work specifically with the population of graduate workers in health. 

Therefore, even though they are developing a dual role, work and study, we still do not know the psychosocial risk factors to which these students are often exposed at work and which can result in illness. In this sense, this research presents the following guiding question: what are the psychosocial risk factors to which master’s and doctoral students who work in the health area are exposed?

This topic is relevant given the impact that psychosocial factors and risks can have on the professional, academic and personal lives of these health workers. This lens, given the different levels of healthcare, is also an original element of this proposal, standing out for comprehensiveness with which psychosocial risk factors faced by working students are analyzed, whether in primary, secondary or tertiary healthcare. Furthermore, knowledge of these risk factors has the potential to contribute to the creation of more welcoming and flexible educational and work settings, and to the formulation of more effective occupational health policies, especially for those who are enrolled in advanced studies.

Given the above, this study aimed to analyze psychosocial risk factors related to the work of graduate students in health.

## METHOD

### Study Design

This is a qualitative, exploratory study, guided by Consolidated Criteria for Reporting Qualitative Research^(10)^.

### Place

The study was conducted in five graduate health programs at a Brazilian public Higher Education Institution (HEI) located in the countryside of the state of São Paulo, Brazil. The programs at the institution under investigation aim to train researchers and healthcare professionals in order to value interdisciplinarity, create knowledge and practices that support social demands, and enable transformation in teaching, research, and healthcare.

### Population

The population was composed of healthcare professionals undertaking *stricto sensu* graduate studies (masters’ and doctoral).

### Selection Criteria

Masters’ and doctoral students enrolled in the five programs of the selected HEI, exercising professional activities in the health area, whether in the public or private sector, enrolled in the programs for more than six months; this time of entry into the program refers to a possible period of student adaptation to the course.

### Data Collection

Data collection was carried out from October to December 2023. Data were obtained through a questionnaire, with sociodemographic, academic and professional information, and, subsequently, a script with guiding questions, using the focus group (FG) technique, according to the foundations proposed by Gatti^(11)^. In addition to the in-person choice, it was decided to carry out the FG technique online due to the flexibility of days and times, considering that it is an audience with multiple attributions.

Regarding the selection techniques in data collection operationalization, the intentional non-probabilistic form (judgment)^(12)^ and also the snowball technique were used, using reference networks and indications^(13)^ based on the inclusion criteria of this study.

Participants were invited in person and remotely through the study’s dissemination by the HEI’ Communication and Multimedia Section, sent to the institutional email of all graduate students. Moreover, they were invited via social media (Instagram^®^) and instant messaging app (WhatsApp^®^). Different selection and approach strategies were used due to the difficulty of graduate students who work in healthcare to participate in the study.

The focus groups (FGs) were attended by the moderator, the main author of this article, and an observer, the second author. The researcher, the FG moderator, has a degree in nursing, a master’s degree and, at the time of data collection, a doctoral candidate. The group observer also has a degree in nursing, a higher education professor, holding a master’s degree, a doctoral degree and a post-doctoral degree. Both have experience and training in the FG technique, are linked to the same HEI and have no conflicts of interest.

The date and time of the groups were defined based on the availability of student workers who agreed to participate. A relationship was established with participants before developing the FGs, through the introduction of the moderator and the observer, as well as their academic and professional backgrounds, personal objectives and the reasons and interests for developing the research.

Concerning the location of the in-person groups, they were held in a private room at the proposing HEI. Remotely, the groups were held via Google Meet^®^, in a private physical room, so as not to interrupt the moderator’s conduct. In both modalities, there were no other people present besides participants and researchers.

Information was audio-recorded with a recorder, and each group lasted an average of 60 minutes. The guiding questions of the study were: “How do you perceive your workplace?” and “What conditions are offered? Are there any difficulties?”. The questions were developed based on literature^(5,6,7,14)^ and researchers’ experience on the subject. Therefore, it was decided not to perform prior script tests, starting directly with the data collection phase. A field diary was also used to record the researchers’ perceptions during the groups. This information was useful for data analysis and was written during and after FGs.

The exact number of participants, as well as the number of FGs carried out, were defined when the power of information was reached, and it was decided to end data collection as soon as the study objective was achieved^(15)^. It is worth noting that there was no need to repeat FGs with the same participants.

### Data Analysis and Treatment

Based on the audio recording of FGs, the reports were transcribed, and the transcripts were returned to participants and validated by participants without adding or reducing the data obtained in groups. Subsequently, information was interpreted according to deductive thematic analysis^(16)^, anchored in the European Agency for Health at Work theoretical framework^(5#x2013;7)^. Thus, the analysis process was carried out manually, without the use of software, following the following stages: familiarization with the data; generation of initial codes; topic search; topic review; topic definition and naming; and report production^(16)^.

It is noteworthy that the analysis phase was carried out by the main researcher, who is experienced and has training in the aforementioned method, completing a *stricto sensu* graduate degree (master’s level) at the same time as working in health. This fact contributed to the process of reflecting on the findings. Furthermore, the entire analysis was carefully reviewed by the other authors of this study.

### Ethical Aspects

The study was approved by the Research Ethics Committee of the proposing institution, under protocol Certificate of Presentation for Ethical Assessment 74404423.0.0000.5393 and approval opinion 6.424.493. Thus, the Informed Consent Form was made available to graduate students in two versions, printed and online.

To preserve participant anonymity, statements were presented with the Arabic number of the FG corresponding to the order in which it was carried out (e.g., FG1, FG2...), followed by participants’ level of education (doctoral degree (D), direct doctoral degree (DD), master’s degree (M), professional master’s degree (PM)), plus the professional category, position and context, if applicable.

Although all participants have degrees in the health field, there are several professional categories, positions and varied contexts. Thus, the final citation was composed of the FG number, the participant’s level of education, profession, role and, if applicable, context of activity: (FG1-D-Nur-Assist-Hosp, FG2-DD-Psycho-Clin, FG4-PM-Nur-Assist-P-Care). It is worth noting that, as there are participants who have multiple employment relationships, for these, the nomenclature was presented between slashes “/” (e.g., FG2-D-Nur-Professor/Management-Cooper). Chart 1 shows the representation of the citation of statements.

**Chart 1:** Nomenclatures used for the names in statements, pre­serving anonymity.

	Acronym	Meaning
**Professional category**	Den-Sur Nur Physical Speech Psycho	Dental Surgeon Nurse Physiotherapist Speech therapist Psychologist
**Role**	Assist Clin Management	Assistance Clinical Manager
**Context**	P-Care Cooper Hosp Health-Depart ECU	Primary Care Cooperative Hospital Health Department Emergency Care Unit

## RESULTS

A total of 364 working graduate students were invited via institutional email by the proposing HEI’s Communication and Multimedia Section. Directly, by the first author, 135 were invited, of which five refused to participate claiming unavailability and 68 did not respond to the invitations. Thus, the study was developed with 62 working *stricto sensu* graduate students, distributed in 13 FGs, 12 remote and one in-person. The in-person FG corresponded to the second (FG2), and the others were remote. Regarding the participants’ profile, the majority were female (n = 45) 72.6% and held a degree in nursing (n = 53) 85.5%, followed by psychology (n = 5) 8.1%, dentistry (n = 2) 3.2%, physical therapy and speech therapy (n = 1) 1.6% each. Regarding the level of the program, they were enrolled in academic doctoral programs (n = 23) 37.1%, academic master’s programs (n = 20) 32.3%, professional master’s programs (n = 12) 19.4%, and direct doctoral programs (n = 7) 11.3%. Concerning the level of professional practice, (n = 12) 19.35% worked in primary care; (n = 24) 38.71% in secondary care; and (n = 26) 41.94% in tertiary care.

### Psychosocial Risk Factors in Healthcare Work

**Figure 1:**
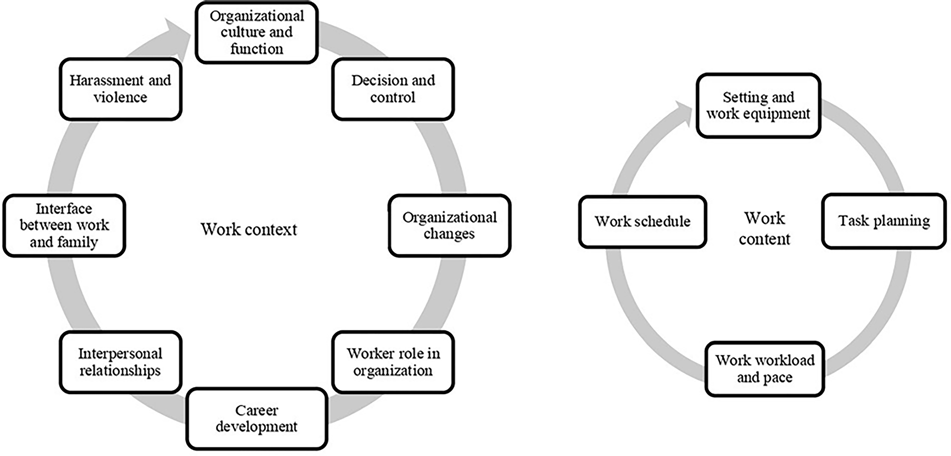
Schematic representation of psychosocial risk factors related to work context and content, organized into empirical categories and subcategories.

The “Psychosocial risk factors in healthcare work” metacategory was constructed, which was subdivided into two main categories, “Work context” and “Work content”, each with its own subcategories. Within “Work context”, the subcategories included the following risk factors: organizational culture and function; decision and control; organizational changes; worker role in organization; career development; interpersonal relationships; interface between work and family; and harassment and violence. Within the scope of “Work content”, the subcategories included the following risk factors: setting and work equipment; task planning; work workload and pace; and work schedule. This form of organization was carried out based on deductive thematic analysis, and is schematically represented in Figure 1.

#### Category 1: Work Context

Work context refers to the physical and social setting in which tasks are performed, which in this study corresponds to health, whether in primary, secondary or tertiary care.

#### Subcategory 1: Organizational Culture and Function

For some graduate health workers, their work setting has an organizational culture where access to managers is difficult, which culminates in a feeling of professional loneliness. There is also authoritarian leadership and a lack of incentives and bonuses, especially in the face of higher education:

*Regarding management, it’s also complicated, it’s not easy. It’s not easy to get access; to get access to them, it’s kind of complicated. So, you kind of have to go it alone* (FG5-D-Nur-Assist-Hosp).

*There are some managements that are very old. So, they have a way of leading* (...) *that is archaic.* (...) *and we end up suffering for wanting to change, because I tried to do something and I was “cut off”* [restricted] (FG6-D-Nur-Assist-Hosp).

*From the institution where I work, zero* [incentive], *in fact I don’t get any financial bonus for the graduate degree. I think that, maybe, if I tell someone, maybe I’ll get congratulations, and that’s it* (FG10-M-Nur-Assist-P-Care).

#### Subcategory 2: Decision and Control

Decision and control refer to limited participation in decision-making and lack of control over tasks. Thus, there are difficulties with flexibility, resulting in a lack of support and personal control at work:

*I have a hard time getting permission, especially when it comes to the sandwich doctorate, you know? And I see that it is more common for nurses, because doctors get permission to go abroad* (FG4-D-Nur-Assist-Hosp).

*I had to take classes, do the course, always during working hours. So, this was extremely difficult for me* (...) *what I feel is the lack of support* (...) *when this professional is taking a graduate course, there is no support whatsoever* (...) *from superiors, from our management* (FG12-M-Nur-Assist-P-Care).

*I have brought ideas and ways for us to solve problems several times, and they say, “Well, we understand that it is a weakness, a necessity, but for the hospital’s administration and board, right now, at the moment, it is not possible”* (FG8-M-Nur-Assist-Hosp).

#### Subcategory 3: Organizational Changes

Changes occurring in the organizational context also prove to be important psychosocial risk factors:

*And there are many changes all the time. Last week was different from this week. So, everything changes very quickly. So, this also ends up demanding a very big emotional issue* (FG6-M-Nur-Management-Cooper).

*We have been having a lot of difficulties in relation to the changes, because everything is new. The documents are all modified, to meet the current needs; from the company’s perspective, to meet hospital policies. So, we are quite overwhelmed* (FG11-D-Nur-Management-Hosp).

*The hospital as a whole is going through changes at the moment, but we also had a change in the leadership of our sector (...) it creates insecurity* (FG1-PM-Nur-Assist-Hosp).

#### Subcategory 4: Worker role in Organization

There is ambiguity in roles and responsibilities at work, especially the duties and pressures of dealing with other people’s lives:

*The pressure itself that I have to handle, I have to be good, because it’s other people’s lives, right? Always someone’s father, someone’s husband who is there in my hands and I have to handle it* (FG8-M-Nur-Assist-Hosp).

*There are situations that happen that I don’t even know how to resolve. I’ll have to research, look, to see how I’m going to provide support to this user* (FG12-D-Nur-Management-P-Care).

*You’re not being paid for what you’re doing, the positions you’re occupying there. And being aware of this (...) this financial devaluation as well* (FG6-DD-Nur-Management-Cooper).

#### Subcategory 5: Career Development

Career development, as a psychosocial risk factor in the work of professionals seeking to qualify through stricto sensu graduate studies, is related to the lack of progression and clarity in their professional trajectory, as well as to a limited perception of the value attributed to their work:

*And they don’t value the title, so I think it’s a little frustrating. They don’t value us who invest in our careers, even though they take advantage of our talent, our writing skills, our methodology* (...) *I feel undervalued* (FG4-D-Nur-Management-Health-Department).

*Sometimes, you only get more work because you’re more qualified* (FG2-D-Nur-Assist-Hosp).

*Where I work, there’s no career plan (...) that’s really lacking, especially the issue of encouraging those who are willing to study to continue their work and financial incentives* (FG4-D-Nur-Assist-Hosp).

#### Subcategory 6: Interpersonal Relationships

Interpersonal relationships can be challenging in some contexts, whether with staff, managers, or users:

*I feel that there is a lack of proper communication* (...) *among the multidisciplinary team. It can cause a lot of discomfort in the work routine; it ends up harming the patient and sometimes it causes stress for the team* (FG2-M-Physical-Assist-Hosp).

*And even the management, immature, has no experience, does not know how to deal with employees, lacks empathy* (FG13-D-Den-Sur-P-Care).

*Dealing with the public is exhausting. When I say public, I include people, right? Both coworkers and users in general* (FG12-D-Nur-Management-P-Care).

#### Subcategory 7: Interface Between Work and Family

Interface between work and family is outlined by a multifaceted journey, requiring the need to balance work, studies, family/personal life and also domestic responsibilities:

*It is a triple journey, including domestic life, hygiene routine, cleaning... it brings a very high level of stress and, at times, a certain exhaustion* (FG13-DD-Psycho-Clin).

*I don’t feel very satisfied with the time I can dedicate to my family, boyfriend, friends; I’ve distanced myself a lot from the friendships I had before for the simple fact that, sometimes, working six days in a row, with classes five days of them, I don’t have the social energy to see anyone* (FG11-M-Nur-Assist-ECU).

*Sometimes, I feel so inadequate that I would like to be at more family gatherings* (...) *it feels like I’m not 100% present* (FG2-M-Speech-Clin).

#### Subcategory 8: Harassment and Violence

Harassment and violence have proven to be risk factors present in healthcare work settings, in addition to discrimination, racism and religious intolerance:

*I work in an Emergency Care Unit, and it is a unit surrounded by a lot of ‘chaos’* [scandal] *and lack of education* (...) *which makes it harder for us to do our job* (FG11-M-Nur-Assist-ECU).

*We have moral violence. Sometimes, the user is unhappy with the service and ends up offending the worker.* (...) *I feel like we are very much on our own when it comes to support. People curse, offend, and that’s it, and we have to get used to that, unfortunately.* (FG12-D-Nur-Management-P-Care).

*In the hospital, I saw what moral harassment is really like* (...) *mainly from the nursing management.* (...) *people come in and use foul language, even insulting people, calling them stupid, that they don’t know anything. And when we go to speak up or report it, we are afraid of being punished. So, we would stay quiet, suffer and not want to talk about it so as not to run the risk of being fired, for instance* (FG6-D-Nur-Assist-Hosp).

*I work directly with managers, directors, so I deal with sensitive issues in the hospital. I have even reached the point where directors asked me to change data in the system, change data on inpatients, because that would interfere with the revenue from funds. Very serious things* (FG3-PM-Nur-Management-Hosp/Professor).

*I suffered religious intolerance and racism. It was also a very challenging time and it shook me throughout the month, even because of the investigation process itself, having to keep reliving it* (FG6-DD-Nur-Management-Cooper).

#### Category 2: Work Content

Work content addresses the intrinsic nature of work activities, covering the following psychosocial risk factors: setting and work equipment; task planning; work workload and pace; and work schedule. These factors will be presented below.

#### Subcategory 1: Setting and Work Equipment

As for setting and work equipment, there are challenges related to reliability, practicality and/or resources for carrying out the work. Some graduate workers face a lack of basic material resources, information systems and limited infrastructure in the health area:

*We are going through a time of great difficulty in terms of material resources, lack of basic materials. We were without adhesive tape and micropore* [microporous tape] (...) *without 100 ml, 250 ml, 500 ml saline solution* (FG1-PM-Nur-Assist-Hosp).

*You have to work with what you have* (FG13-PM-Nur-Assist-Hosp).

*The computer has a very low processing speed. The internet goes down, the power sometimes goes out, you know? The nearest bathroom is very far away. There is no sink, for instance, to wash your hands, you know? It is an organ adapted within a larger structure* (FG12-PM-Nur-Management-Hosp).

#### Subcategory 2: Task Planning

Task planning corresponds to the lack of opportunities to acquire new knowledge. There is frustration due to the lack of guidance and training by the coordination and administration in certain work contexts. But there is also devaluation by workers, where there is a reluctance to participate in lectures and training to acquire knowledge:

*Things come up and we have to do them without any training, without any qualifications for it. It’s very exhausting* (FG10-M-Nur-Assist-Hosp).

*Generally, we receive a lot of vulnerable population, homeless people, a very high psychiatric demand. So, there are many professionals who don’t have the “touch”* [training] *with this vulnerable population* (FG11-PM-Nur-Assist-ECU).

*We notice that it’s even difficult to train these employees, because they are never available for training* (FG11-D-Nur-Management-Hosp).

#### Subcategory 3: Work Workload and Pace

It is worth noting that workload and pace are also emphasized in healthcare, including in self-employment:

*Even in the management area, it is a very demanding area. You have to be very present; unlike, for instance, a medical coordinator. The medical coordinator has the position, but they do not work “full time” in the sector. Today, a nursing coordinator needs to be there* (FG10-M-Nur-Management-Hosp).

*What makes it difficult, sometimes, is the issue of overwork, which is practically inherent to the nursing profession, taking over many responsibilities* (FG12-M-Nur-Assist-Hosp).

*So, there is a lot of work that I take home to set up this therapy, to write the report, to set up the protocols (...) there is no one to take care of my schedule (...) the issuing of notes. So, I also do all this bureaucratic part* (FG2-M-Speech-Clin).

#### Subcategory 4: Work Schedule

Work schedule includes unpredictable hours, long working hours and night shifts. The lack of fixed hours and the need to cover different shifts can adversely impact the routine and time available for activities outside of work, such as graduate studies. This leads to complexity and instability in the time management of these professionals:

*On weekends, we are occasionally called upon, as is management* (FG1-D-Nur-Management-Health-Depart/Professor).

*I don’t have a fixed schedule, I’m kind of a day off, so I work morning, afternoon, night, when needed, and sometimes this affects my graduate activities* (FG2-D-Nur-Assist-Hosp).

*I work at night. So, sometimes, there will be times when I sleep the whole day... an extremely tired sleep, a sleep that doesn’t rest* (FG11-M-Nur-Assist-ECU).

*I work at night, and during the day, we are very tired* (FG13-PM-Nur-Assist-Hosp).

## DISCUSSION

According to the analysis of psychosocial risk factors related to the work of graduate students in health in this research, it was noted that working graduate students face significant psychosocial risk factors in the context of health work, which, consequently, influence their emotional reactions, with an impact not only on work, but extending to other areas of life, including academic life.

Thus, it is important to note that psychosocial risk factors may be related to work context, within the scope of organizational culture and function as well as its management practices. Harmful behavior by a leader is associated with psychological distress among employees, which results in negative effects ranging from loss of self-esteem, withdrawal, restlessness, avoidance to concern and resistance to providing feedback and contributions at work^(17)^. Qualitative research, carried out in Norway with master nurses, revealed that participants’ experiences are ambivalent, as these professionals are threats at the same time as they are also resources in work organization^(18)^.

It was observed that many healthcare professionals, especially in the healthcare field, face a lack of autonomy at work. From this perspective, when there is a favorable work setting where employees enjoy adequate autonomy, they feel empowered and motivated to reach their maximum potential^(5)^, which contrasts with the results of this study, since workers feel unmotivated.

In relation to resources, this study highlighted limitations, including emotional support for workers. Integrative and Complementary Health Practices^(1)^, the implementation of initiatives focused on self-management, employee empowerment and access to mental healthcare services, such as mindfulness or meditation sessions, in the workplace, can contribute to reducing levels of psychological stress, anxiety and burnout among professionals^(19)^, and are therefore relevant for this population.

However, the lack of availability of these resources, as well as work overload, or other risk factors present in work context and content can generate negative impacts on worker health. Moreover, the ways of dealing with work dynamics and structure in health are challenging and can result in significant illness among professionals. This context is aggravated by the complexity of working in healthcare, combined with the demands of managing academic responsibilities, which also involve challenges and requirements^(9)^. Such conditions, as they are structured, constitute potential sources of illness.

Although mental health and quality of life at work, especially among health workers, are largely affected by organizational and occupational structures, which are managed by both organizations and employees themselves, there is limited attention to capitalist labor relations that perpetuate the unhealthy conditions of the contemporary work setting. In this context, it is the responsibility of management initiatives and training centers to address mental health at work, including issues such as exploitation, alienation and resistance within the capitalist system^(20)^, including psychosocial risk factors.

Regarding decision and control, this research clarified that the lack of flexibility and support for academic activities and professional development can limit workers’ autonomy in managing their time and developing their skills. Hence, managing work schedules, especially in hospital settings, is challenging due to the conflict between employers’ needs and workers’ desires for stability and predictability. Factors such as perceived control, supervisor support, shift changes, and overtime affect emotional exhaustion and turnover intentions^(21)^.

A study conducted in China suggests that greater organizational support can also improve research engagement, strengthening researchers’ intrinsic and extrinsic motivation. Thus, universities and hospitals have the potential to provide education on the importance of health research, which supports intrinsic and extrinsic motivation for research. For nurses in master’s programs, hospitals can adjust policies according to their needs, providing organizational support to facilitate the research learning process^(22)^. However, in practice, there are barriers to organizational support that can impact the involvement of graduate workers in their studies and, consequently, their engagement.

With regard to organizational changes, it has been found that, when they occur, can affect workers psychosocially. Thus, poorly managed or poorly communicated changes within an organization can cause stress due to the uncertainty and doubt they generate in people^(5)^.

In relation to organizational roles, ambiguities in roles and responsibilities have been reported and are important psychosocial risk factors in the work context. Therefore, it is necessary to ensure that workers have a clear understanding of their roles and the responsibilities of others involved in work^(5)^.

Regarding career development, this research observed a lack of career recognition and appreciation, especially of the academic title. A study conducted online with radiology students and professionals from the clinical and academic team on a global scale, obtaining 176 responses, found that a doctoral qualification is not necessary for career progression for 126(72%) respondents^(23)^. These findings are similar to what this study shows, since, for some participants, the degree is not considered for career progression, which generates a feeling of demotivation and lack of recognition by work organization^(9)^.

As for the interface between work and family, in recent decades, Western countries have undergone significant demographic, cultural and social changes that have affected the interface between work and family. These transformations have highlighted the challenges of simultaneously balancing professional, family and domestic responsibilities^(24)^. Thus, the goal is to balance work and personal life in order to create a more harmonious integration between the professional and personal spheres, but it is a significant barrier and, in this sense, a psychosocial risk factor.

This study showed that interpersonal relationships correspond to another psychosocial risk factor arising from different relationships, whether with coworkers or managers. In the context of healthcare organizations, interpersonal relationships are essential. German researchers propose that structural measures, such as regular team meetings and appropriate training, are useful for improving interprofessional communication in a specific way^(25)^. Hence, in addition to self-control and emotional balance of those involved, it is important to have leaders capable of intervening and negotiating conflicts in favor of resolution and harmony at work.

The presence of moral harassment and violence at work was another psychosocial risk factor identified in this research, the first being mainly due to differences in treatment and duties, as well as offensive terms, insults and coercion to perform unethical practices, generating harm and moral suffering, in addition to racism and intolerance. In this line of thought, healthcare professions involve intense daily contact with people, especially patients, coworkers and managers. These interactions also have the potential to expose these workers to situations of moral harassment/bullying and violence, risk factors with serious consequences for those involved^(26)^.

Regarding work content, one of the psychosocial risk factors highlighted was work setting and equipment, which in this study corresponded especially to the lack of supplies, deficient infrastructure and outdated information systems. Thus, maintaining adequate working conditions is essential to preserve the well-being and good performance of workers, especially nurses^(27)^.

Concerning task planning, this study found a lack of guidance and training as initiatives of workplace management. However, from another perspective, according to some managers participating in this research, it was noted that some workers undervalued training and qualifications. This fact may be due to workers’ reluctance or unavailability to participate in lectures and training that would be beneficial for professional practice^(28)^.

In terms of high workload and pace, some workers can cope with very demanding tasks where many challenges and pressures are placed on them. However, these demands can cause stress if the person feels that they cannot cope with them or do not have enough control over them. Some workers will have difficulty admitting to having problems due to too many demands, perhaps seeing it as a sign of weakness or inadequacy^(5)^. Furthermore, in healthcare, the workload of nursing professionals is highlighted, which directly affects the quality of care and the psychosocial quality of these workers.

This study found that workers face variable schedules, covering different shifts and periods of on-call, resulting in unpredictable and long working hours, including night work. Companies often adopt practices with low wages and long working hours to maximize productivity, which can adversely affect worker health^(29)^. This fact, in addition to interfering with mental health, can also impact physical health, with the literature demonstrating a negative association between night work and cardiovascular health^(30)^.

It is therefore important to recognize the ever-changing nature of work in the 21^st^ century and to make efforts to develop effective interventions to help overcome these problems^(29)^, with an emphasis on psychosocial risks and the factors involved. It is necessary to develop intervention proposals and/or public policies that promote healthy work settings, especially in healthcare, and in conjunction with HEIs, especially when workers have a workday that is simultaneous with graduate studies.

As limitations of this study, it should be considered that it was conducted with health workers who were also *stricto sensu* graduate students from a Brazilian public HEI. Furthermore, there was a predominance of nurses and a shortage of other professional categories and data collection in both in-person and remote formats. However, as strengths, it should be considered the presence of participants working in institutions from different contexts (primary, secondary and tertiary healthcare), which lends credibility to the scope of psychosocial risk factors existing in health work.

As for the advances of this study for the areas of health and nursing, managers have the legal responsibility to ensure adequate risk assessment and control. Furthermore, the participation of workers in this process is pertinent, in order to ensure the identification and more effective management of problems, in addition to being a necessary topic for educational interventions and incorporation into the curricular matrix of HEIs in health.

For future studies, new research is suggested, especially with a qualitative approach with this student-worker audience, with the inclusion of professionals from different categories in the health area, in order to broaden the understanding of psychosocial risk factors at work. Moreover, it is recommended to carry out longitudinal studies that allow the analysis of the evolution of these factors over time as well as research that explores coping strategies and interventions for promoting worker mental health.

## CONCLUSION

The analysis of psychosocial risk factors related to the work of graduate students in health reflects the particularities of each work context and content, organizations and levels of care, with emphasis on the factors presented in nurses’ work, which also have the potential to significantly influence academic engagement. Therefore, it is necessary that such factors, in addition to being managed in work settings, be incorporated and deepened in the educational programs of training institutions. This implies integrating these topics into the curricular matrix of undergraduate and graduate courses, with a view to developing/improving skills, raising awareness and preparing future professionals to deal with the psychosocial challenges and dangers that may arise in their careers, whether in primary, secondary or tertiary healthcare.

## DATA AVAILABILITY

Available data. The entire dataset supporting the results of this study was published in the article itself.
