# Supplementary material for: Psychosocial risk factors at work: perspectives of master’s and doctoral students working in the health sector
Source: Rev Esc Enferm USP. 2025 Jul 7;59:e20250045. doi: 10.1590/1980-220X-REEUSP-2025-0045en (PMC12245393; doi:10.1590/1980-220X-REEUSP-2025-0045en)
Supplement: Translation

# Translation: Fatores de risco psicossociais no trabalho: perspectivas de mestrandos e doutorandos trabalhadores na área da saúde*

**DOI:** 10.1590/1980-220X-REEUSP-2025-0045pt

**Authors:** Carolina Cassiano, Laura Andrian Leal, Elisabete Maria das Neves Borges, José Carlos Marques de Carvalho, Silvia Helena Henriques

**Affiliations:** 1 Universidade de São Paulo, Escola de Enfermagem de Ribeirão Preto, Ribeirão Preto, SP, Brasil.; 2 Universidade de São Paulo, Escola de Enfermagem de Ribeirão Preto, Departamento de Enfermagem Geral e Especializada, Ribeirão Preto, SP, Brasil.; 3 Escola Superior de Enfermagem do Porto, Porto, Portugal.

**Keywords:** Riscos Ocupacionais, Saúde Ocupacional, Pessoal de Saúde, Estudantes, Programas de Pós-Graduação em Saúde

## Abstract

**Objetivo:**

Analisar os fatores de risco psicossociais relacionados ao trabalho de pós-graduandos atuantes na área da saúde.

**Método:**

Estudo qualitativo exploratório, com mestrandos e doutorandos trabalhadores na área da saúde, vinculados a cinco programas de pós-graduação de uma instituição de ensino superior pública brasileira. A coleta de dados ocorreu entre outubro e dezembro de 2023, utilizando questionário sociodemográfico, acadêmico e profissional, e grupos focais guiados por roteiro. Utilizou-se análise temática dedutiva para interpretação dos dados.

**Resultados:**

Participaram 62 pós-graduandos trabalhadores. Identificaram-se os seguintes fatores de risco psicossociais no contexto do trabalho: cultura e função organizacional; decisão e controle; mudanças organizacionais; função do trabalhador na organização; desenvolvimento de carreira; interface entre trabalho e família; relacionamento interpessoal; assédio e violência. No conteúdo do trabalho, os fatores foram ambiente e equipamentos de trabalho, planejamento das tarefas, carga e ritmo, e esquema de trabalho.

**Conclusão:**

Centros formadores e organizações de trabalho devem se atentar para os fatores de risco psicossociais aos quais estão expostos os seus pós-graduandos trabalhadores, de modo que sejam incorporadas estratégias para o seu gerenciamento.

## INTRODUÇÃO

O trabalho nas organizações de saúde se constrói por meio da articulação de processos distintos, nos quais cada profissional contribui com seus conhecimentos e instrumentos próprios de atuação. No Brasil, tem-se a atenção primária, que oferece cuidados básicos, preventivos e de gestão de condições crônicas, sendo a porta de entrada para o Sistema Único de Saúde, focada no acompanhamento contínuo. A atenção secundária envolve serviços especializados, como consultas com especialistas e alguns exames mais complexos. Já a atenção terciária é voltada para tratamentos avançados, como cirurgias de alta complexidade e cuidados intensivos. Vale ressaltar que cada nível de atenção visa garantir cuidados adequados conforme a necessidade do paciente^(1)^.

Tais modalidades de atenção, assim como as organizações de saúde, dependem cada vez mais de equipes de pesquisa integradas para otimizar a prestação de cuidados de alta qualidade baseados em evidências que melhoram a experiência assistencial^(2)^. Portanto, os trabalhadores da saúde devem ser capacitados para atender às necessidades cotidianas de saúde das pessoas. Tal fato se estende para a pós-graduação *stricto sensu*, sendo que há um crescimento desses trabalhadores se candidatando a esses programas, com vistas à qualificação e desenvolvimento de pesquisas^(3)^.

Desse modo, tem-se a contribuição com o desenvolvimento científico em saúde, o qual pode impactar positivamente a assistência. Outrossim, os cuidados em saúde são complexos e diversificados, e cada profissão dessa área tem características únicas e necessárias para a complementação assistencial. No entanto, o trabalho, especificamente na área da saúde, a despeito de ser um contexto propício para a formação de identidade, significados psíquicos e interações sociais, também pode resultar em riscos e adoecimento^(4)^.

Nessa perspectiva, os riscos psicossociais no trabalho referem-se a falhas na organização e na gestão do contexto ou do conteúdo laboral, capazes de provocar prejuízos psicológicos, físicos e sociais ao trabalhador^(5#x2013;7)^. Tais riscos podem ser desencadeados pelos fatores de risco psicossociais, como sobrecarga de trabalho, ausência de clareza nas atribuições profissionais, baixa participação nas decisões, má gestão de mudanças, precariedade laboral, comunicação ineficaz, falta de apoio, assédio e interação com pessoas difíceis, como revela o referencial teórico adotado para este estudo^(5,6)^.

A crescente atenção aos fatores de risco psicossociais, aos riscos psicossociais e suas potenciais consequências na saúde dos trabalhadores ganhou destaque devido às transformações no mundo do trabalho. As mudanças recentes nesse cenário global intensificaram os fatores de risco psicossociais existentes e introduziram novos a serem considerados. Isso incita a abertura de debates, tanto em âmbito nacional quanto em âmbito internacional, sobre a necessidade de priorizar esta problemática nas políticas, estratégias e ações relacionadas ao trabalho^(8)^.

Articulando essa problemática com a população da pós-graduação, investigadores do Reino Unido evidenciam que muitos estudantes de doutorado experimentam decepção e estresse durante seus estudos, muitas vezes relacionados à falta de apoio e aos desafios de concluir o curso enquanto trabalham^(9)^.

Embora os cursos de pós-graduação na área da saúde sejam imprescindíveis para a produção de conhecimento, no atendimento às demandas de saúde, além de possibilitarem a qualificação dos profissionais no âmbito do trabalho, tem-se uma lacuna científica quanto aos fatores de risco psicossociais no trabalho especificamente com a população de pós-graduandos trabalhadores da área da saúde.

Portanto, mesmo desenvolvendo dupla atribuição, trabalho e estudo, ainda não se sabem os fatores de risco psicossociais aos quais esses estudantes, muitas vezes, estão expostos no trabalho e que podem resultar em adoecimento. Nessa direção, esta pesquisa apresenta a seguinte questão norteadora: quais os fatores de risco psicossociais aos quais estão expostos os mestrandos e doutorandos que trabalham na área da saúde?

Esta temática se faz pertinente diante do impacto que os fatores e os riscos psicossociais podem assumir na vida profissional, acadêmica e pessoal desses trabalhadores da saúde. Essa lente, pelos diferentes níveis de atenção à saúde, também é um elemento de originalidade desta proposta, destacando- se pela abrangência com que se analisam os fatores de risco psicossociais enfrentados por estudantes trabalhadores, seja na atenção primária, secundária e terciária à saúde. Outrossim, o conhecimento desses fatores de risco tem potencial de contribuir para a criação de ambientes educacionais e trabalhistas mais acolhedores e flexíveis, e para a formulação de políticas de saúde ocupacionais mais profícuas, sobretudo para aqueles que estão inseridos em estudos avançados.

Diante do exposto, o objetivo deste estudo é analisar os fatores de risco psicossociais relacionados ao trabalho de pós- graduandos atuantes na área da saúde.

## MÉTODO

### Desenho do Estudo

Trata-se de um estudo qualitativo, exploratório, norteado pelo guia de verificação *Consolidated Criteria for Reporting Qualitative Research* ^(10)^.

### Local

O estudo foi desenvolvido em cinco programas de pós-graduação da saúde de uma instituição de ensino superior (IES) pública brasileira, localizada no interior do estado de São Paulo, Brasil. Os programas da instituição investigada têm o intuito de formar pesquisadores e profissionais de saúde, de modo a valorizar a interdisciplinaridade, criar conhecimento e práticas que corroborem as demandas sociais, bem como possibilitar a transformação no ensino, pesquisa e cuidado em saúde.

### População

A população foi composta por profissionais de saúde realizando pós-graduação *stricto sensu* (mestrado e doutorado).

### Critérios de Seleção

Mestrandos e doutorandos matriculados nos cinco programas da IES selecionada, exercendo atividades profissionais na área da saúde, seja no setor público ou privado, matriculados nos programas há mais de seis meses; este tempo de entrada no programa se refere a um possível período de adaptação do estudante ao curso.

### Coleta de Dados

A coleta de dados foi realizada de outubro a dezembro de 2023. Os dados foram obtidos por meio de um questionário, com informações sociodemográficas, acadêmicas e profissionais, e, posteriormente, um roteiro com questões norteadoras, utilizando-se a técnica de grupo focal (GF), conforme os fundamentos propostos por Gatti^(11)^. Além da escolha presencial, optou-se por realizar a técnica de GF *online* em virtude da flexibilidade de dias e horários, considerando ser um público com múltiplas atribuições.

Quanto às técnicas de seleção na operacionalização da coleta de dados, utilizaram-se a forma não probabilística intencional (julgamento)^(12)^ e também a técnica *snowball* ou bola de neve, utilizando redes de referência e indicações^(13)^ com base nos critérios de inclusão deste estudo.

Os participantes foram convidados presencialmente e remotamente por meio da divulgação do estudo pela Seção de Comunicação e Multimeios da IES, direcionada ao e-mail institucional de todos os pós-graduandos. Ademais, foram convidados por rede social (*Instagram* ^®^) e por aplicativo de mensagens instantâneas (*WhatsApp* ^®^). As diferentes estratégias de seleção e abordagem foram utilizadas devido à dificuldade de adesão dos estudantes de pós-graduação trabalhadores da saúde com o estudo.

Os Grupos Focais (GFs) tiveram a participação da moderadora, autora principal deste artigo, e de uma observadora, segunda autora. A pesquisadora, moderadora dos GFs, é graduada em enfermagem, tem titulação em mestrado e, na época da coleta de dados, doutoranda. A observadora dos grupos também é graduada em enfermagem, professora do magistério superior, mestra, doutora e pós-doutora. Ambas têm experiência e treinamento na técnica de GF, vinculadas à mesma IES e sem conflitos de interesse.

A data e horário dos grupos foram definidos a partir da disponibilidade dos estudantes trabalhadores que aceitaram participar. Estabeleceu-se um relacionamento com os participantes antes do desenvolvimento dos GFs, por meio da apresentação da moderadora e da observadora, bem como suas formações acadêmicas e profissionais, objetivos pessoais e as razões e interesses para desenvolver a pesquisa.

Em relação ao local de realização dos grupos presencialmente, realizou-se em sala reservada na IES proponente. Pela via remota, os grupos foram realizados pelo *Google Meet* ^®^, em uma sala física reservada, para não haver interrupções na condução da moderadora. Em ambas as modalidades, não havia outras pessoas presentes além dos participantes e pesquisadoras.

As informações foram audiogravadas com gravador, e cada grupo teve duração média de 60 minutos. As perguntas norteadoras do estudo foram “Como você percebe o seu local de trabalho?” e “Quais condições são oferecidas? Existem dificuldades?”. As questões foram elaboradas com base na literatura^(5#x2013;7,14)^ e na experiência dos pesquisadores sobre a temática. Assim, optou-se por não realizar testes prévios do roteiro, iniciando-se diretamente a fase de coleta de dados. Utilizou-se também um diário de campo para que fossem registradas as percepções das pesquisadoras no decorrer dos grupos. Essas informações foram úteis para a análise de dados e foram redigidas durante e após os GFs.

A quantidade exata de participantes, bem como o número de GFs realizados, foram definidos quando o poder da informação foi atingido, optando-se por encerrar a coleta de dados assim que o objetivo do estudo foi alcançado^(15)^. Ressalta-se que não houve necessidade de repetição de GFs com os mesmos participantes.

### Análise e Tratamento dos Dados

A partir da gravação em formato de áudio dos GFs, foram transcritos os relatos, sendo realizada a devolução das transcrições aos participantes, sendo validadas por eles, sem haver acréscimos ou redução dos dados obtidos nos grupos. Posteriormente, as informações foram interpretadas conforme a análise temática dedutiva^(16)^, ancorada no referencial teórico da European Agency for Health at Work^(5–7)^. Assim, o processo de análise foi realizado manualmente, sem o uso de *software*, seguindo as seguintes etapas: familiarização com os dados; geração de códigos iniciais; busca por temas; revisão dos temas; definição e nomeação dos temas; e produção do relatório^(16)^.

Sobressalta-se que a fase de análise foi realizada pela pesquisadora principal, experiente e com formação e treinamento no método supracitado, realizando a pós-graduação *stricto sensu* (nível mestrado) concomitante ao trabalho em saúde. Este fato contribuiu para o processo de reflexividade dos achados. Ademais, toda a análise foi cuidadosamente revisada pelos demais autores deste estudo.

### Aspectos Éticos

O estudo foi aprovado pelo Comitê de Ética em Pesquisa da instituição proponente, sob protocolo Certificado de Apresentação para Apreciação Ética n^o^ 74404423.0.0000.5393 e parecer de aprovação n^o^ 6.424.493. Assim, foi disponibilizado para os pós-graduandos o Termo de Consentimento Livre e Esclarecido em duas versões, impresso e *online*.

Para preservar o anonimato dos participantes, os depoimentos foram apresentados com o número arábico do GF, correspondendo à ordem em que foi realizado (*e.g*., GF1, GF2...), seguido do nível de formação do participante (doutorado (D), doutorado direto (DD), mestrado (M), mestrado profissional (MP)), acrescido da categoria profissional, cargo e contexto, se aplicável.

Embora todos os participantes sejam graduados na área da saúde, há diversas categorias profissionais, cargos e contextos variados. Assim, a citação final foi composta pelo número do GF, o nível de formação do participante, profissão, função e, se aplicável, contexto de atuação: (GF1-D-Enf-Assist-Hosp, GF2-DD-Psico-Clín, GF4-MP-Enf-Assist-At-Primária). Destaca-se que, como há participantes que possuem múltiplos vínculos, para estes, foi apresentada a nomenclatura entre barra “/” (*e.g*., GF2-D-Enf-Docente/Gestão-Cooper). Tem-se a representação da citação dos depoimentos no Quadro 1.

**Quadro 1:** Nomenclaturas utilizadas para as denominações nos depoimentos, preservando o anonimato.

	Sigla	Significado
**Categoria profissional**	Cirur-Dent Enf Fisio Fono Psico	Cirurgião Dentista Enfermeiro Fisioterapeuta Fonoaudiólogo Psicólogo
**Função**	Assist Clín Gestão	Assistencial Clínico Gestor
**Contexto**	At-Primária Cooper Hosp Secret-Saúde UPA	Atenção Primária Cooperativa Hospitalar Secretaria de Saúde Unidade de Pronto Atendimento

## RESULTADOS

Um total de 364 pós-graduandos trabalhadores foram convidados via e-mail institucional pela Seção de Comunicação e Multimeios da IES proponente. Diretamente, pela primeira autora, foram convidados 135, sendo que cinco recusaram participar alegando indisponibilidade e 68 não responderam aos convites. Assim, o estudo foi desenvolvido com 62 estudantes de pós-graduação *stricto sensu* trabalhadores, distribuídos em 13 GFs, sendo 12 remotos e um presencial. O GF presencial correspondeu ao segundo (GF2), e os demais foram remotos. Quanto ao perfil dos participantes, a maioria do gênero feminino (n = 45) 72,6% e graduada em enfermagem (n = 53) 85,5%, seguida por psicologia (n = 5) 8,1%, odontologia (n = 2) 3,2%, fisioterapia e fonoaudiologia (n = 1) 1,6% cada. Quanto ao nível de curso, cursavam o doutorado acadêmico (n = 23) 37,1%, o mestrado acadêmico (n = 20), 32,3%, o mestrado profissional (n = 12), 19,4% e o doutorado direto (n = 7), 11,3%. Em relação ao nível de atuação dos participantes, (n = 12) 19,35% atuantes na atenção primária; (n = 24) 38,71% na secundária e (n = 26) 41,94% na terciária.

### Fatores de Risco Psicossociais no Trabalho em Saúde

Construiu-se a metacategoria “Fatores de risco psicossociais no trabalho em saúde”, devido à amplitude de dados, a qual foi subdividida em duas categorias principais, “Contexto do trabalho” e “Conteúdo do trabalho”, cada uma com suas próprias subcategorias. No âmbito do “Contexto do trabalho”, as subcategorias incluíram os seguintes fatores de risco: cultura e função organizacional; decisão e controle; mudanças organizacionais; função do trabalhador na organização; desenvolvimento de carreira; interface entre trabalho e família; relacionamento interpessoal; e assédio e violência. No âmbito do “Conteúdo do trabalho”, as subcategorias incluíram os seguintes fatores de risco: ambiente e equipamentos de trabalho; planejamento das tarefas; carga e ritmo de trabalho; e esquema de trabalho. Essa forma de organização foi realizada a partir da análise temática dedutiva, e está representada esquematicamente na Figura 1.

**Figura 1:** Representação esquemática dos fatores de risco psicossociais relacionados ao contexto e ao conteúdo do trabalho, organizados em categorias e subcategorias empíricas.

#### Categoria 1: Contexto do Trabalho

O contexto do trabalho se refere ao ambiente físico e social no qual as tarefas são realizadas, que neste estudo corresponde à saúde, seja na atenção primária, secundária ou terciária.

#### Subcategoria 1: Cultura e Função Organizacional

Para alguns pós-graduandos trabalhadores da saúde, o ambiente de trabalho possui uma cultura organizacional onde o acesso aos gestores é complexo, o que culmina em uma sensação de solidão profissional. Tem-se também a liderança autoritária e a carência de incentivos e bonificações, sobretudo diante da titulação:

*Em relação à chefia que é complicado também, não é uma coisa fácil. Não são fáceis o acesso; pra você ter acesso a eles, é meio complicado. Então, você meio que anda sozinho* (GF5-D-Enf-Assist-Hosp).

*Existem algumas chefias que são muito antigas. Então, eles têm um jeito de liderar* (...) *que é arcaico.* (...) *e a gente acaba sofrendo por querer mudar, porque eu tentava fazer alguma coisa e eu era “podado”* [cerceado] (GF6-D-Enf-Assist-Hosp).

*Por parte da instituição que eu trabalho, zero* [incentivo]*, inclusive eu não tenho nenhuma gratificação financeira pela pós-graduação. Eu acho que, talvez, se eu falar para alguém, talvez eu ganhe um parabéns, e olhe lá* (GF10-M-Enf-Assist-At-Primária).

#### Subcategoria 2: Decisão e Controle

A decisão e controle se refere à participação limitada na tomada de decisão e ausência de controle sobre as tarefas. Assim, tem-se dificuldades quanto à flexibilidade, resultando em falta de apoio e controle pessoal no trabalho:

*Eu sinto uma dificuldade por conta de liberação mesmo, ainda mais na questão do doutorado sanduíche, sabe? E eu vejo que é mais com a classe de enfermeiros, porque a classe médica consegue liberação para ir para o exterior* (GF4-D-Enf-Assist-Hosp).

*Eu tinha que cursar as aulas, fazer a disciplina, sempre em horário de trabalho. Então, isso foi uma dificuldade extrema para mim* (...) *o que eu sinto é a falta de apoio* (...) *quando esse profissional está cursando a pós-graduação, não tem nenhum tipo de apoio* (...) *dos superiores, da nossa gestão* (GF12-M-Enf-Assist-At-Primária).

*Várias vezes, já levei ideias, maneiras da gente resolver os problemas, e eles falam: “Ai, a gente entende que é uma fragilidade, uma necessidade, mas para o administrativo e diretoria do hospital, agora, no momento, não é possível”* (GF8-M-Enf-Assist-Hosp).

#### Subcategoria 3: Mudanças Organizacionais

As mudanças ocorridas no contexto organizacional também se mostram fatores de risco psicossocial importantes:

*E acontecem muitas mudanças a todo momento. Semana passada, já é diferente dessa semana. Então, tudo muda muito rápido. Então, isso também acaba demandando uma questão emocional muito grande* (GF6-M-Enf-Gestão-Cooper).

*A gente vem tendo bastante dificuldades em relação às mudanças, porque é tudo novo. Os documentos são todos modificados, para atender às necessidades atuais; na perspectiva da empresa, para atender às políticas hospitalares. Então, a gente fica bastante sobrecarregada* (GF11-D-Enf-Gestão-Hosp).

*O hospital como um todo está passando por mudanças nesse momento, mas a gente teve uma mudança de chefia do nosso setor também* (...) *gera insegurança* (GF1-MP-Enf-Assist-Hosp).

#### Subcategoria 4: Função do Trabalhador na Organização

Tem-se a ambiguidade de funções e responsabilidades no trabalho, especialmente as incumbências e pressões ao lidar com a vida de outras pessoas:

*A pressão por si só que eu tenho que dar conta, eu tenho que ser boa, porque é a vida de outras pessoas, né? Sempre o pai de alguém, marido de alguém que está ali nas minhas mãos e eu tenho que dar conta* (GF8-M-Enf-Assist-Hosp).

*Tem situações que acontecem que eu nem sei como resolver. Vou ter que pesquisar, procurar, para ver como que eu vou dar um apoio para esse usuário* (GF12-D-Enf-Gestão-At-Primária).

*Você não ser remunerado por aquilo que você está desenvolvendo, os cargos que você está ocupando ali dentro. E ter noção disso* (...) *dessa desvalorização também financeira* (GF6-DD-Enf-Gestão-Cooper).

#### Subcategoria 5: Desenvolvimento de Carreira

O desenvolvimento de carreira, como fator de risco psicossocial no trabalho desses profissionais que buscam se qualificar por meio da pós-graduação stricto sensu, está relacionado à ausência de progressão e clareza na trajetória profissional, bem como à visão limitada do valor atribuído ao trabalho.

*E eles não valorizam o título, então acho que é um pouco frustrante. A gente que investe na nossa carreira, eles não valorizam, apesar de aproveitarem muito do nosso talento, de escrever, de ter metodologia* (...) *eu me sinto desvalorizado* (GF4-D-Enf-Gestão-Secret-Saúde).

*Às vezes, você só consegue mais trabalho por ser mais qualificado* (GF2-D-Enf-Assist-Hosp).

*Onde eu trabalho, não tem plano de carreira* (...) *falta muito isso, principalmente a questão de incentivar quem está disposto a estudar a levar para frente o trabalho e o incentivo financeiro* (GF4-D-Enf-Assist-Hosp).

#### Subcategoria 6: Relacionamento Interpessoal

O relacionamento interpessoal pode ser desafiador em alguns contextos, seja com a equipe, gestores e usuários:

*Eu sinto que falta comunicação adequada* (...) *entre a equipe multiprofissional. Pode trazer muito desconforto na rotina de trabalho mesmo, acaba prejudicando o paciente e às vezes gera estresse para a equipe* (GF2-M-Fisio-Assist-Hosp).

*E até mesmo a chefia, imatura, não tem experiência, não sabe lidar com os funcionários, sem empatia* (GF13-D-Cirur-Dent-At-Primária).

*O lidar com o público é desgastante. Quando eu falo público, eu incluo pessoas, né? Tanto colegas de trabalho quanto usuários em geral* (GF12-D-Enf-Gestão-At-Primária).

#### Subcategoria 7: Interface Entre Trabalho e Família

A interface entre trabalho e família é delineada por uma jornada multifacetada, requerendo a necessidade de equilibrar trabalho, estudos, vida familiar/pessoal e também as responsabilidades domésticas:

*É uma jornada tripla, contando a vida doméstica, rotina de higiene, limpeza... traz um nível de estresse muito elevado e, em alguns momentos, uma certa exaustão* (GF13-DD-Psico-Clín)*.*

*Eu não me sinto muito satisfeita com o tempo que eu consigo me dedicar à família, namorado, amigos; me distanciei muito das minhas relações da amizade que eu tinha antes pelo simples fato de que, às vezes, trabalhando seis dias seguidos, com aula cinco dias deles, não tenho energia social para ver ninguém* (GF11-M-Enf-Assist-UPA).

*Às vezes, me bate muito essa insuficiência que eu gostaria de estar em mais reuniões familiares* (...) *parece que eu não estou 100% presente.* (GF2-M-Fono-Clín).

#### Subcategoria 8: Assédio e Violência

O assédio e violência se mostraram fatores de risco presentes no ambiente de trabalho em saúde, além da discriminação, do racismo e da intolerância religiosa:

*Eu trabalho em uma Unidade de Pronto Atendimento, e é uma unidade cercada por muito ‘barraco’* [escândalo] *e falta de educação* (...) *o que torna mais difícil a gente fazer a nossa atividade* (GF11-M-Enf-Assist-UPA).

*Temos violência moral. Às vezes, o usuário está descontente com um atendimento e ele acaba ofendendo o trabalhador.* (...) *eu sinto que a gente fica muito sozinho no sentido de respaldo. As pessoas xingam, ofendem, e pronto, e a gente tem que estar acostumado a isso, infelizmente.* (GF12-D-Enf-Gestão-At-Primária).

*No hospital, eu vi o que é a assédio moral mesmo* (...) *principalmente da gerência de enfermagem.* (...) *da pessoa chegar e usar termos de baixo calão, xingar mesmo, de burro, que não sabe. E quando a gente vai falar ou denunciar, a gente tem medo de ser punido. Então, a gente ficava quieto, sofrendo e não queria falar para não ter o perigo de ser demitido, por exemplo* (GF6-D-Enf-Assist-Hosp).

*Eu trabalho diretamente com gestores, diretores, então lido com questões sensíveis do hospital. Eu já cheguei ao ponto de diretores me pedirem pra alterar dados no sistema, alterar dados de pacientes internados, porque aquilo ia interferir na receita de verbas. Coisas bem pesadas* (GF3-MP-Enf-Gestão-Hosp/Docente).

*Eu sofri intolerância religiosa e racismo. Foi um momento muito desafiador também e me abalou durante o mês todo, até pelo processo de apuração mesmo, de ter que ficar revivenciando* (GF6-DD-Enf-Gestão-Cooper).

#### Categoria 2: Conteúdo do Trabalho

O conteúdo do trabalho aborda a natureza intrínseca das atividades laborais, abrangendo os seguintes fatores de risco psicossociais: ambiente e equipamentos de trabalho; planejamento das tarefas; carga e ritmo de trabalho; e esquema de trabalho. Esses fatores serão apresentados a seguir.

#### Subcategoria 1: Ambiente e Equipamentos de Trabalho

No que se refere ao ambiente e equipamentos de trabalho, tem-se desafios relacionados à confiabilidade, praticidade e/ou recursos para a execução do trabalho. Alguns pós-graduandos trabalhadores enfrentam a falta de recursos materiais básicos, sistemas de informação e infraestrutura limitados na área da saúde:

*A gente está passando por um momento de grande dificuldade em relação a recursos materiais, falta de material básico. A gente estava sem esparadrapo e sem micropore* [fita microporosa] (...) *sem soro fisiológico de 100 ml, de 250 ml, de 500 ml* (GF1-MP-Enf-Assist-Hosp).

*Você tem que ir trabalhando com o que tem* (GF13-MP-Enf-Assist-Hosp).

*O computador é uma velocidade de processamento muito ruim. A internet cai, a luz às vezes cai, entendeu? O banheiro mais próximo é bem distante. Não tem pia, por exemplo, para lavar a mão, entendeu? É um órgão adaptado dentro de uma estrutura maior* (GF12-MP-Enf-Gestão-Hosp).

#### Subcategoria 2: Planejamento das Tarefas

O planejamento de tarefas corresponde à carência de oportunidades para aquisição de novos conhecimentos. Há frustração devido à falta de orientação e capacitação por parte da coordenação e administração em determinados contextos de trabalho. Mas tem-se também a desvalorização por parte do trabalhador, onde reside uma relutância em participar de palestras e treinamentos para adquirir conhecimento:

*As coisas aparecem e nós temos que fazer sem nenhuma capacitação, sem nenhum treinamento para isso. É bem desgastante* (GF10-M-Enf-Assist-Hosp).

*Geralmente, a gente recebe muita população vulnerável, situação de rua, uma demanda psiquiátrica muito grande. Então, são muitos profissionais que não têm “tato”* [capacitação] *com essa população vulnerável* (GF11-MP-Enf-Assist-UPA).

*A gente observa que até para capacitar esses funcionários é difícil, porque eles nunca estão disponíveis para capacitação* (GF11-D-Enf-Gestão-Hosp).

#### Subcategoria 3: Carga e Ritmo de Trabalho

Vale ressaltar que a carga e ritmo de trabalho também são enfatizados na assistência em saúde, incluindo no trabalho autônomo:

*Mesmo pra área de gestão, é uma área que é muito cobrada. Você tem que estar muito presente; diferente, por exemplo, de um coordenador médico. O coordenador médico, ele tem o cargo, mas ele não fica “full time”* [o tempo todo] *no setor. Hoje, um coordenador de enfermagem, ele precisa estar* (GF10-M-Enf-Gestão-Hosp).

*O que dificulta, às vezes, é a questão do excesso de trabalho, que é praticamente inerente da profissão dos enfermeiros, assumindo muitas responsabilidades* (GF12-M-Enf-Assist-Hosp).

*Então, tem muito trabalho que eu levo para casa de montar terapia, de fazer o relatório, de montar os protocolos* (...) *não tem uma pessoa que cuida da minha agenda* (...) *a emissão de notas. Então, toda essa parte burocrática eu também faço* (GF2-M-Fono-Clín).

#### Subcategoria 4: Esquema de Trabalho

O esquema de trabalho abarca horários imprevisíveis, extensas jornadas laborais e turnos noturnos. A falta de horários fixos e a necessidade de cobrir diferentes turnos podem impactar adversamente a rotina e o tempo disponível para atividades fora do trabalho, como no caso dos estudos de pós-graduação. Isso aduz à complexidade e instabilidade na gestão do tempo desses profissionais:

*Os finais de semana, eventualmente a gente é acionado, como é gestão* (GF1-D-Enf-Gestão-Secret-Saúde/Docente).

*Eu não tenho horário fixo, eu sou meio que uma folguista, então eu trabalho de manhã, tarde, noite, quando precisa, e aí isso às vezes dá uma prejudicada nas atividades da pós* (GF2-D-Enf-Assist-Hosp).

*Eu trabalho noturno. Então, às vezes, vão ter horários que eu vou dormir o dia inteiro... um sono de extremo cansaço também, um sono que não descansa* (GF11-M-Enf-Assist-UPA).

*Eu trabalho à noite, e, durante o dia, a gente está muito cansado* (GF13-MP-Enf-Assist-Hosp).

## DISCUSSÃO

De acordo com a análise dos fatores de risco psicossociais relacionados ao trabalho de pós-graduandos atuantes na área da saúde, nesta pesquisa, percebeu-se que os estudantes de pós-graduação trabalhadores enfrentam fatores de risco psicossociais significativos no âmbito do trabalho em saúde, os quais, consequentemente, influenciam suas reações emocionais, com impacto não só no trabalho, mas se estendendo para outras áreas da vida, inclusive a acadêmica.

Assim, faz-se relevante abordar que os fatores de risco psicossociais podem estar relacionados ao contexto de trabalho, no âmbito da cultura e função organizacional, bem como suas práticas de gestão. O comportamento nocivo de um líder está associado ao sofrimento psicológico dos funcionários, o que resulta em efeitos negativos, os quais abrangem desde a perda de autoestima, reclusão, inquietação, evitação até a preocupação e resistência em fornecer *feedback* e contribuições no trabalho^(17)^. Uma pesquisa qualitativa, realizada na Noruega com enfermeiros mestres, revelou que as experiências dos participantes são ambivalentes, pois esses profissionais são ameaças ao mesmo tempo em que também são recursos na organização de trabalho^(18)^.

Observou-se que muitos profissionais de saúde, sobretudo no campo assistencial, enfrentam falta de autonomia no trabalho. Nessa perspectiva, quando existe um ambiente laboral propício onde os colaboradores desfrutam de autonomia adequada, sentem-se capacitados e motivados para alcançar suas potencialidades^(5)^, o que contrasta com os resultados deste estudo, uma vez que alguns trabalhadores se sentem desmotivados.

No que concerne aos recursos, este estudo evidenciou limitações, inclusive de apoio emocional para os trabalhadores. As Práticas Integrativas e Complementares em Saúde^(1)^, a implementação de iniciativas centradas na autogestão, o empoderamento dos colaboradores e o acesso a serviços de saúde mental, como sessões de *mindfulness* ou meditação, no ambiente de trabalho, podem contribuir para a diminuição dos níveis de estresse psicológico, ansiedade e esgotamento entre os profissionais^(19)^, sendo, portanto, relevantes para esta população.

No entanto, a falta de disponibilidade desses recursos, assim como a sobrecarga de trabalho, ou outros fatores de risco presentes no contexto e conteúdo do trabalho podem gerar prejuízos à saúde do trabalhador. Além disso, as formas de enfrentar a dinâmica e a estrutura do trabalho na saúde são desafiadoras e podem resultar em adoecimento significativo dos profissionais. Esse contexto se agrava pela complexidade de atuar na assistência em saúde, somada às exigências de gerenciar as demandas acadêmicas, as quais também apresentam dificuldades e exigências^(9)^. Tais condições, como estão estruturadas, configuram-se como fontes potenciais de adoecimento.

Embora a saúde mental e a qualidade de vida no trabalho, especialmente entre os trabalhadores da saúde, sejam afetadas majoritariamente pelas estruturas organizacionais e ocupacionais, que são gerenciadas tanto pelas organizações quanto pelos próprios funcionários, há atenção limitada às relações de trabalho capitalistas que perpetuam as condições insalubres do ambiente profissional contemporâneo. Nesse contexto, é responsabilidade das iniciativas de gestão e dos centros formadores abordar a saúde mental no trabalho, incluindo questões como exploração, alienação e resistência dentro do sistema capitalista^(20)^, incluindo os fatores de risco psicossociais.

No que concerne à decisão e controle, esta pesquisa esclareceu que a falta de flexibilidade e apoio para atividades acadêmicas e aprimoramento profissional pode limitar a autonomia dos trabalhadores em gerenciar seu tempo e desenvolver suas habilidades. Nesse sentido, gerenciar os horários de trabalho, especialmente no âmbito hospitalar, é desafiador, devido ao conflito entre as necessidades dos empregadores e os desejos dos trabalhadores por estabilidade e previsibilidade. Fatores como controle percebido, apoio do supervisor, mudanças de turno e horas extras afetam a exaustão emocional e as intenções de rotatividade^(21)^.

Um estudo realizado na China alude que maior apoio organizacional pode aprimorar inclusive o envolvimento na pesquisa e fortalecer a motivação intrínseca e extrínseca dos pesquisadores. Desse modo, universidades e hospitais têm o potencial de oferecer educação sobre a importância da pesquisa em saúde, o que corrobora a motivação intrínseca e extrínseca para a investigação. Para enfermeiros em programas de mestrado, os hospitais podem ajustar políticas conforme suas necessidades, concedendo apoio organizacional para facilitar o processo de aprendizagem da pesquisa^(22)^. No entanto, na prática, há barreiras quanto ao suporte organizacional que podem impactar inclusive o envolvimento dos pós-graduandos trabalhadores com os estudos e, consequentemente, seu engajamento.

No que tange às mudanças organizacionais, verificou-se que, quando ocorrem, podem afetar os trabalhadores psicossocialmente. Desse modo, as mudanças mal gerenciadas ou mal comunicadas dentro de uma organização podem causar estresse devido à incerteza e dúvida que geram nas pessoas^(5)^.

Em relação às funções organizacionais, ambiguidades de funções e responsabilidades foram relatadas e são importantes fatores de risco psicossociais no contexto de trabalho. Portanto, é preciso garantir que os trabalhadores tenham compreensão clara de seus papéis e das responsabilidades dos outros envolvidos no trabalho^(5)^.

No que tange ao desenvolvimento de carreira, observou-se nesta investigação a falta de reconhecimento, valorização da carreira, especialmente do título acadêmico. Um estudo desenvolvido *online* com estudantes de radiologia e profissionais da equipe clínica e acadêmica em escala global, obtendo 176 respostas, verificou que a qualificação de doutoramento não é necessária para a progressão na carreira para 126 (72%) inquiridos^(23)^. Esses achados se assemelham ao que evidencia este estudo, uma vez que, para alguns participantes, a titulação não é considerada para progressão na carreira, o que gera o sentimento de desmotivação e falta de reconhecimento por parte da organização de trabalho^(9)^.

No que tange à interface entre trabalho e família, nas últimas décadas, os países ocidentais passaram por mudanças demográficas, culturais e sociais relevantes que afetaram a interface entre trabalho e família. Essas transformações destacaram os desafios para equilibrar simultaneamente as responsabilidades profissionais, familiares e domésticas^(24)^. Assim, busca-se a conciliação entre trabalho e vida pessoal para conceber uma integração mais harmoniosa entre as esferas profissional e pessoal, porém é uma barreira importante e, nessa direção, um fator de risco psicossocial.

Este estudo mostrou que o relacionamento interpessoal corresponde a outro fator de risco psicossocial derivado de relacionamentos diversos, seja com colegas de trabalho ou gestores. No contexto das organizações de saúde, as relações interpessoais são imprescindíveis. Pesquisadores alemães propõem que medidas estruturais, como reuniões regulares de equipe e formações adequadas, são úteis para aprimorar a comunicação interprofissional de maneira específica^(25)^. Nessa linha de pensamento, além do autocontrole e equilíbrio emocional dos envolvidos, faz-se relevante haver líderes capazes de intervir e negociar conflitos em prol da resolutividade e harmonia no trabalho.

A presença de assédio moral e violência no trabalho foi outro fator de risco psicossocial identificado nesta pesquisa, sendo o primeiro oriundo principalmente de distinção de tratamento e atribuições, bem como termos ofensivos, xingamentos e coação para a realização e práticas antiéticas, gerando dano e sofrimento moral, além de racismo e intolerância. Nessa linha de pensamento, as profissões da área da saúde abarcam um contato diário intenso com pessoas, com destaque para os pacientes, colegas de trabalho e gestores. Essas interações também têm potencial de expor esses trabalhadores a situações de assédio moral/*bullying* e violência, fatores de risco com consequências graves para os envolvidos^(26)^.

Em relação ao conteúdo do trabalho, um dos fatores de risco psicossocial destacados foram os ambientes e equipamentos de trabalho, que neste estudo corresponderam especialmente à falta de insumos, infraestrutura deficitária e sistemas de informação ultrapassados. Dessa forma, a manutenção de condições de trabalho adequadas é fundamental para preservar o bem-estar e bom desempenho dos trabalhadores, com destaque para os enfermeiros^(27)^.

No que concerne ao planejamento de tarefas, percebeu-se neste estudo falta de orientação e capacitação como iniciativas da gestão do local de trabalho. No entanto, sob outra perspectiva, percebeu-se desvalorização de capacitações e treinamentos por parte de alguns trabalhadores, de acordo com alguns gestores participantes desta pesquisa. Tal fato pode ser decorrente da relutância ou indisponibilidade dos trabalhadores em participar de palestras e treinamentos que seriam profícuos para a prática profissional^(28)^.

No tocante à carga e ritmo de trabalho elevados, alguns trabalhadores podem lidar com tarefas muito exigentes, onde muitos desafios e pressões são colocados sobre eles. No entanto, essas demandas podem causar estresse se a pessoa sentir que não consegue lidar com elas ou se não tiver controle suficiente sobre elas. Alguns trabalhadores terão dificuldade em admitir ter problemas devido a muitas demandas, talvez considerando isso como um sinal de fraqueza ou inadequação^(5)^. Além disso, na saúde, ressalta-se a sobrecarga de trabalho da enfermagem, a qual interfere diretamente na qualidade do cuidado e na qualidade psicossocial desses trabalhadores.

Este estudo identificou que os trabalhadores enfrentam horários variáveis, cobertura de diferentes turnos e períodos de sobreaviso, resultando em jornadas de trabalho imprevisíveis e prolongadas, incluindo o trabalho noturno. Empresas frequentemente adotam práticas com salários baixos e jornadas longas para maximizar a produtividade, o que pode afetar adversamente a saúde dos trabalhadores^(29)^. Tal fato, além de interferir na saúde mental, também pode prejudicar a saúde física, sendo evidenciada na literatura a associação negativa entre trabalho noturno e saúde cardiovascular^(30)^.

É relevante, portanto, reconhecer a natureza em constante transformação do trabalho no século XXI e empreender esforços para desenvolver intervenções eficazes que ajudem a superar esses problemas^(29)^, com destaque para os riscos psicossociais e os fatores que os envolvem. Faz-se necessário desenvolver propostas de intervenção e/ou políticas públicas que promovam ambientes de trabalho saudáveis, sobretudo na saúde e articulados com as IES, sobretudo quando os trabalhadores têm jornada de trabalho simultânea com os estudos de pós-graduação.

Como limitações deste estudo, deve-se considerar que foi realizado com trabalhadores da saúde também estudantes de pós-graduação *stricto sensu* de uma IES pública brasileira. Além disso, houve predomínio de enfermeiros, escassez de outras categorias profissionais e coleta de dados nos formatos presencial e remoto. No entanto, como pontos fortes, deve-se considerar a presença de participantes atuantes em instituições de contextos diversos (atenção primária, secundária e terciária em saúde), o que confere a credibilidade da abrangência dos fatores de risco psicossociais existentes no trabalho em saúde.

Em relação aos avanços deste estudo para as áreas da saúde e enfermagem, os gestores têm a responsabilidade legal de garantir a avaliação e o controle adequados dos riscos. Ademais, a participação dos trabalhadores nesse processo é pertinente, de modo a assegurar a identificação e gestão mais eficaz dos problemas, além de ser uma temática necessária para intervenções educativas e incorporação na matriz curricular de IES em saúde.

Sugerem-se, para estudos futuros, novas investigações, sobretudo com a abordagem qualitativa com este público estudante trabalhador, com a inclusão de profissionais de diversas categorias da área da saúde, de modo a ampliar a compreensão dos fatores de risco psicossociais no trabalho. Além disso, recomenda-se a realização de estudos longitudinais que possibilitem a análise da evolução desses fatores ao longo do tempo, bem como pesquisas que explorem estratégias de enfrentamento e intervenções para a promoção da saúde mental dos trabalhadores.

## CONCLUSÃO

A análise dos fatores de risco psicossociais relacionados ao trabalho de pós-graduandos atuantes na área da saúde reflete as particularidades de cada contexto e conteúdo do trabalho, organizações e níveis de atenção, com destaque para os fatores apresentados no trabalho dos enfermeiros, os quais também têm potencial de influenciar significativamente o engajamento acadêmico. Logo, é necessário que tais fatores, além de geridos no ambiente de trabalho, sejam incorporados e aprofundados nos programas educacionais das instituições formadoras. Isso implica integrar esses temas à matriz curricular dos cursos de graduação e de pós-graduação, com vistas a desenvolver/aprimorar competências, conscientizar e preparar os futuros profissionais para lidar com os desafios e perigos psicossociais que podem surgir em suas carreiras, seja na atenção primária, secundária ou terciária à saúde.

## DISPONIBILIDADE DE DADOS

Dados disponíveis. Todo o conjunto de dados que dá suporte aos resultados deste estudo foi publicado no próprio artigo.

# Translation: Factores de riesgo psicosocial en el trabajo: perspectivas de estudiantes de maestría y doctorado que trabajan en el sector salud*

**DOI:** 10.1590/1980-220X-REEUSP-2025-0045es

**Authors:** Carolina Cassiano, Laura Andrian Leal, Elisabete Maria das Neves Borges, José Carlos Marques de Carvalho, Silvia Helena Henriques

**Affiliations:** 1 Universidade de São Paulo, Escola de Enfermagem de Ribeirão Preto, Ribeirão Preto, SP, Brasil.; 2 Universidade de São Paulo, Escola de Enfermagem de Ribeirão Preto, Departamento de Enfermagem Geral e Especializada, Ribeirão Preto, SP, Brasil.; 3 Escola Superior de Enfermagem do Porto, Porto, Portugal.

**Keywords:** Riesgos Laborales, Salud Laboral, Personal de Salud, Estudiantes, Programas de Posgrado en Salud

## Abstract

**Objetivo:**

Analizar los factores de riesgo psicosocial relacionados con el trabajo de estudiantes de posgrado que actúan en el área de la salud.

**Método:**

Estudio cualitativo exploratorio, con estudiantes de maestría y doctorado que actúan en el área de la salud, vinculados a cinco programas de posgrado de una institución pública de educación superior brasileña. La recolección de datos se realizó entre octubre y diciembre de 2023, mediante un cuestionario sociodemográfico, académico y profesional, y grupos focales guiados por un guión. Para interpretar los datos se utilizó el análisis temático deductivo.

**Resultados:**

Participaron 62 estudiantes de posgrado en activo. Se identificaron los siguientes factores de riesgo psicosocial en el contexto laboral: cultura y función organizacional; decisión y control; cambios organizacionales; función del trabajador en la organización; desarrollo profesional; interfaz entre trabajo y familia; relación interpersonal; acoso y violencia. En el contenido del trabajo los factores fueron ambiente y equipo de trabajo, planificación de tareas, carga y ritmo, y esquema de trabajo.

**Conclusión:**

Los centros de formación y las organizaciones laborales deben prestar atención a los factores de riesgo psicosocial a los que están expuestos sus trabajadores de posgrado, de manera que se puedan incorporar estrategias para su gestión.

## INTRODUCCIÓN

El trabajo en las organizaciones de salud se construye mediante la articulación de diferentes procesos, en los que cada profesional aporta sus conocimientos y herramientas. En Brasil, la atención primaria ofrece cuidados básicos, prevención y gestión de enfermedades crónicas, y es la puerta de entrada al Sistema Único de Salud, centrándose en el seguimiento continuo. La atención secundaria incluye servicios especializados, como consultas con especialistas y algunas pruebas más complejas. La atención terciaria se centra en tratamientos avanzados, como cirugías de alta complejidad y cuidados intensivos. Merece la pena destacar que cada nivel de atención pretende garantizar una asistencia adecuada a las necesidades del paciente^(1)^.

Estos tipos de asistencia, así como las organizaciones sanitarias, dependen cada vez más de los equipos de investigación integrados para optimizar la prestación de una asistencia de alta calidad basada en la evidencia que mejore la experiencia asistencial^(2)^. Por lo tanto, el personal sanitario debe estar formado para satisfacer las necesidades sanitarias cotidianas de las personas. Esto se extiende a los estudios de posgrado *stricto sensu*, donde se observa un aumento de estos trabajadores que solicitan estos programas con vistas a la cualificación y el desarrollo de la investigación^(3)^.

Ello contribuye al desarrollo científico en salud, que puede repercutir positivamente en la asistencia. Además, la atención en salud es compleja y diversa, y cada profesión en esta área tiene características únicas y necesarias para complementar la atención. Sin embargo, el trabajo, concretamente en el sector sanitario, a pesar de ser un contexto favorable para la formación de la identidad, los significados psíquicos y las interacciones sociales, también puede dar lugar a riesgos y enfermedades^(4)^.

Desde esta perspectiva, los riesgos psicosociales en el trabajo se refieren a fallos en la organización y gestión del contexto o contenido del trabajo, capaces de causar daños psicológicos, físicos y sociales al trabajador^(5–7)^. Estos riesgos pueden ser desencadenados por factores de riesgo psicosocial como la sobrecarga de trabajo, la falta de claridad en las atribuciones profesionales, la escasa participación en la toma de decisiones, la mala gestión del cambio, las condiciones de trabajo precarias, la comunicación ineficaz, la falta de apoyo, el acoso y la interacción con personas difíciles, como revela el marco teórico adoptado para este estudio^(5,6)^.

La creciente atención a los factores de riesgo psicosocial, a los riesgos psicosociales y a sus posibles consecuencias para la salud de los trabajadores ha ganado protagonismo debido a las transformaciones del mundo laboral. Los cambios recientes en este escenario global han intensificado los factores de riesgo psicosocial existentes e introducido otros nuevos a tener en cuenta. Esto ha suscitado debates tanto a escala nacional como internacional sobre la necesidad de dar prioridad a esta cuestión en las políticas, estrategias y acciones relacionadas con el trabajo^(8)^.

Vinculando esta cuestión a la población de postgraduados, investigadores del Reino Unido han demostrado que muchos estudiantes de doctorado experimentan decepción y estrés durante sus estudios, a menudo relacionados con la falta de apoyo y con los retos que supone completar el curso mientras se trabaja^(9)^.

Aunque los cursos de posgrado en el área de la salud sean esenciales para producir conocimiento, atender a las demandas sanitarias y capacitar a los profesionales en el lugar de trabajo, existe un vacío científico en relación a los factores de riesgo psicosocial en el trabajo, específicamente con la población de estudiantes de posgrado que trabajan en el área de la salud. Por lo tanto, aunque estén trabajando y estudiando, aún se desconocen los factores de riesgo psicosocial a los que estos estudiantes suelen estar expuestos en el trabajo y que pueden resultar en enfermedades. Teniendo esto en cuenta, esta investigación plantea la siguiente pregunta guía: ¿a qué factores de riesgo psicosocial están expuestos los estudiantes de maestría y doctorado que trabajan en el área de la salud?

Esta cuestión es pertinente dado el impacto que los factores y riesgos psicosociales pueden tener en la vida profesional, académica y personal de estos trabajadores de la salud. Este enfoque, a través de los diferentes niveles de atención sanitaria, es también un elemento de la originalidad de esta propuesta, que destaca por el alcance con el que se analizan los factores de riesgo psicosocial a los que se enfrentan los estudiantes trabajadores, ya sea en la atención sanitaria primaria, secundaria o terciaria. Además, el conocimiento de estos factores de riesgo tiene el potencial de contribuir a la creación de entornos educativos y laborales más acogedores y flexibles, y a la formulación de políticas de salud laboral más fructíferas, especialmente para quienes cursan estudios superiores.

Teniendo en cuenta lo anterior, el objetivo de este estudio fue analizar los factores de riesgo psicosocial relacionados con el trabajo de los estudiantes de postgrado que trabajan en el ámbito de la salud.

## MÉTODO

### Diseño del Estudio

Se trata de un estudio cualitativo, exploratorio, guiado por la guía de verificación *Consolidated Criteria for Reporting Qualitative Research* ^(10)^.

### Sitio del Estudio

El estudio se realizó en cinco programas de posgrado en salud de una institución pública brasileña de enseñanza superior (IES) localizada en el interior del estado de São Paulo, Brasil. Los programas de la institución investigada tienen como objetivo formar investigadores y profesionales en salud para valorizar la interdisciplinariedad, crear conocimientos y prácticas que corroboren las demandas sociales y posibiliten la transformación de la enseñanza, la investigación y la atención en salud.

### Población

La población estuvo constituida por profesionales de salud que cursan programas de posgrado *stricto sensu* (maestría y doctorado).

### Criterios de Selección

Estudiantes de maestría y doctorado inscritos en los cinco programas de la IES seleccionada, que trabajen en el sector salud, ya sea en el sector público o privado, inscritos en los programas por más de seis meses; este tiempo de ingreso al programa se refiere a un posible periodo de adaptación del estudiante al curso.

### Recolección de Datos

Los datos se recogieron entre octubre y diciembre de 2023. Los datos se recogieron mediante un cuestionario con información sociodemográfica, académica y profesional, y posteriormente un guión con preguntas orientadoras, mediante la técnica de grupo focal (GF), según los fundamentos propuestos por Gatti^(11)^. Además de la elección presencial, optamos por realizar la técnica de GF *online* debido a la flexibilidad de días y horarios, considerando que se trata de un público con múltiples atribuciones.

En cuanto a las técnicas de selección utilizadas para operacionalizar la recolección de datos, se utilizó de forma intencional no probabilística (juicio)^(12)^ y también la técnica *de bola de nieve*, utilizando redes de referencia e indicios^(13)^ con base en los criterios de inclusión de este estudio.

Se invitó a los participantes de forma presencial y a distancia mediante la divulgación del estudio por parte de la Sección de Comunicación y Multimedia de la IES, enviada a las direcciones de correo electrónico institucionales de todos los estudiantes de posgrado. También se les invitó a través de redes sociales (*Instagram* ^®^) y aplicaciones de mensajería instantánea (*WhatsApp* ^®^). Las diferentes estrategias de selección y acercamiento se utilizaron debido a la dificultad de conseguir que los estudiantes de posgrado de trabajadores de la salud participaran en el estudio.

A los grupos focales (GF) asistieron el moderador, el autor principal de este artículo, y un observador, el segundo autor. La investigadora, que moderó los grupos focales, es diplomada en enfermería, tiene un máster y, en el momento de la recogida de datos, era estudiante de doctorado. El observador de los grupos también es diplomado en enfermería, profesor de enseñanza superior, máster, doctorado y posdoctorado. Ambas tienen experiencia y formación en la técnica de GF, están vinculadas a la misma IES y no tienen conflictos de intereses.

La fecha y la hora de los grupos se fijaron en función de la disponibilidad de los estudiantes trabajadores que aceptaron participar. Se estableció una relación con los participantes antes de que tuvieran lugar los grupos de discusión, presentando al moderador y al observador, así como sus antecedentes académicos y profesionales, sus objetivos personales y las razones e intereses para llevar a cabo la investigación.

Los grupos se celebraron cara a cara en una sala reservada de la IES proponente. Los grupos se celebraron a distancia a través de *Google Meet* ^®^, en una sala física reservada, para no interrumpir al moderador. En ambos casos, no había otras personas presentes aparte de los participantes y los investigadores.

La información se grabó en audio con una grabadora y cada grupo duró una media de 60 minutos. Las preguntas que guiaron el estudio fueron «¿Cómo percibe su lugar de trabajo?» y «¿Qué condiciones se ofrecen? ¿Hay dificultades?». Las preguntas se basaron en la bibliografía^(5–7,14)^ y en la experiencia de los investigadores sobre el tema. Por lo tanto, se decidió no probar el guión de antemano, sino empezar directamente con la fase de recogida de datos. También se utilizó un diario de campo para registrar las percepciones de los investigadores durante los grupos. Esta información fue útil para analizar los datos y se anotó durante y después de los grupos de discusión.

El número exacto de participantes, así como el número de grupos de discusión realizados, se definió cuando se alcanzó la potencia de la información, optándose por finalizar la recogida de datos en cuanto se alcanzó el objetivo del estudio^(15)^. Cabe destacar que no fue necesario repetir las entrevistas con los mismos participantes.

### Análisis y Tratamiento de Los Datos

A partir de la grabación de audio de los grupos de discusión, se transcribieron los informes, y las transcripciones se devolvieron a los participantes y fueron validadas por ellos, sin ninguna adición o reducción de los datos obtenidos en los grupos. Posteriormente, la información se interpretó según el análisis temático deductivo^(16)^, anclado en el marco teórico de la European Agency for Health at Work (Agencia Europea para la Salud en el Trabajo)^(5#x2013;7)^. Así, el proceso de análisis se realizó manualmente, sin la utilización de *software*, siguiendo las siguientes etapas: familiarización con los datos; generación de códigos iniciales; búsqueda de temas; revisión de temas; definición y denominación de temas; y elaboración del informe^(16)^.

Cabe destacar que la fase de análisis fue realizada por la investigadora principal, con experiencia y formación en el método mencionado, realizando sus estudios de posgrado (*nivel de* maestría) simultáneamente con su trabajo en salud. Esto contribuyó a la reflexividad de los resultados. Además, todo el análisis fue cuidadosamente revisado por los demás autores de este estudio.

### Aspectos Éticos

El estudio fue aprobado por el Comité de Ética en Investigación de la institución proponente, bajo el protocolo Certificado de Sometimiento a Evaluación Ética no. 74404423.0.0000.5393 y dictamen de aprobación no. 6.424.493. Así, el Formulario de Consentimiento Libre e Informado se puso a disposición de los posgraduados en dos versiones, impresa y en línea. Para preservar el anonimato de los participantes, los testimonios se presentaron con el número arábigo del GF, correspondiente al orden en que se realizó (por ejemplo, GF1, GF2...), seguido del nivel de formación del participante (doctorado (D), doctorado directo (DD), maestría (M), maestría profesional (MP)), además de la categoría profesional, cargo y contexto, en su caso.

Aunque todos los participantes son licenciados en el ámbito sanitario, existen diferentes categorías profesionales, cargos y contextos. Así, la citación final se compuso del número de GF, nivel de formación del participante, profesión, función y, si procede, contexto en el que trabaja: (GF1-D-Enf-Asistente-Hosp, GF2-DD-Psico-Clin, GF4-MP-Enf-Asistente-At-Primária). Cabe señalar que, como hay participantes que tienen varios vínculos, se les ha dado la nomenclatura con una barra «/» (por *ejemplo,* GF2-D-Enf-Docente/Gestión-Cooper). En el Cuadro 1 se muestra una representación de las declaraciones.

**Cuadro 1:** Nomenclaturas utilizadas para los nombres en los testimonios, preservando el anonimato.

	Sigla	Significado
**Categoría profesional**	Cirur-Dent Enf Fisio Fono Psico	Cirujano Dentista Enfermero Fisioterapeuta Fonoaudiólogo Psicólogo
**Función**	Assist Clín Gestión	Asistencial Clínico Gestor
**Contexto**	At-Primaria Cooper Hosp Secret-Salud UPA	Atención Primaria Cooperativa Hospitalar Secretaría de Salud Unidad de Pronta Atención

## RESULTADOS

La Sección de Comunicación y Multimedia del IES proponente invitó por correo electrónico institucional a un total de 364 postgraduados en activo. El primer autor invitó directamente a 135. Cinco se negaron a participar alegando no estar disponibles y 68 no respondieron a las invitaciones. Así pues, el estudio se llevó a cabo con 62 estudiantes de posgrado en activo, divididos en 13 grupos de discusión, 12 a distancia y uno *presencial*. El GF presencial correspondió al segundo (GF2), y los demás fueron remotos. En cuanto al perfil de los participantes, la mayoría eran mujeres (n = 45) 72,6% y graduadas en enfermería (n = 53) 85,5%, seguidas de psicología (n = 5) 8,1%, odontología (n = 2) 3,2%, fisioterapia y logopedia (n = 1) 1,6% cada una. En cuanto al nivel de titulación, el 37,1% cursaba estudios de doctorado académico (n = 23), el 32,3% de máster académico (n = 20), el 19,4% de máster profesional (n = 12) y el 11,3% de doctorado directo (n = 7). En cuanto al nivel de ejercicio profesional de los participantes, (n = 12) el 19,35% trabajaba en atención primaria, (n = 24) el 38,71% en atención secundaria y (n = 26) el 41,94% en atención terciaria.

### Factores de Riesgo Psicosocial en el Trabajo en Salud

La metacategoría «Factores de riesgo psicosocial en el trabajo en salud» se construyó debido a la amplitud de los datos, que se subdividieron en dos categorías principales, «Contexto laboral» y «Contenido laboral», cada una con sus propias subcategorías. En «Contexto laboral», las subcategorías incluían los siguientes factores de riesgo: cultura y función organizativas; decisión y control; cambios organizativos; papel del trabajador en la organización; desarrollo profesional; interfaz entre trabajo y familia; relaciones interpersonales; y acoso y violencia. En «Contenido del trabajo», las subcategorías incluían los siguientes factores de riesgo: entorno y equipos de trabajo; planificación de tareas; carga y ritmo de trabajo; y esquema de trabajo. Esta forma de organización se basó en el análisis temático deductivo y se representa esquemáticamente en la Figura 1.

**Figura 1:** Representación esquemática de los factores de riesgo psicosocial relacionados con el contexto y el contenido del trabajo, organizados en categorías y subcategorías empíricas.

#### Categoría 1: Contexto Laboral

El contexto laboral se refiere al entorno físico y social en el que se desarrollan las tareas, que en este estudio corresponde a la sanidad, ya sea en atención primaria, secundaria o terciaria.

#### Subcategoría 1: Cultura y Función de la Organización

Para algunos postgraduados sanitarios, el entorno de trabajo tiene una cultura organizativa en la que el acceso a los gestores es complejo, lo que culmina en un sentimiento de soledad profesional. También existe un liderazgo autoritario y una falta de incentivos y bonificaciones, especialmente en relación con las cualificaciones:

*En relación con la gestión, también es complicado, no es fácil. No es fácil acceder a ellos; es un poco complicado para ti acceder a ellos. Así que estás un poco por tu cuenta* (GF5-D-Enf-Assist-Hosp).

*Hay algunos directores que son muy mayores. Entonces tienen una forma de dirigir* (...) *que es arcaica* (...) *y terminamos sufriendo por querer cambiar, porque yo intenté hacer algo y me «podaron* » [cortaron] (GF6-D-Enf-Assist-Hosp).

*Por parte de la institución para la que trabajo, hay cero* [incentivo]*, ni siquiera recibo recompensa económica por mis estudios de posgrado. Creo que si se lo digo a alguien, a lo mejor me felicitan, y* nada más (GF10-M-Enf-Assist-At-Primária).

#### Subcategoría 2: Decisión y Control

Decisión y control se refiere a la participación limitada en la toma de decisiones y a la falta de control sobre las tareas. Esto conlleva dificultades en términos de flexibilidad, lo que se traduce en una falta de apoyo y de control personal en el trabajo:

*Lo veo difícil por la liberación, más aún con el doctorado en alternancia, ¿sabes? Y veo que es más con las enfermeras, porque los médicos pueden obtener permiso para ir al extranjero* (GF4-D-Enf-Assist-Hosp).

*Yo tenía que asistir a las clases, hacer el curso, siempre en horario laboral. Así que fue muy difícil para mí* (...) *lo que siento es la falta de apoyo* (...) *cuando este profesional está haciendo estudios de posgrado, no recibe ningún apoyo* (...) *de sus superiores, de nuestra dirección* (GF12-M-Enf-Assist-At-Primária).

*Varias veces he propuesto ideas, formas de resolver problemas, y me han dicho: «Ah, entendemos que es una debilidad, una necesidad, pero para la administración y la dirección del hospital, ahora mismo, no es posible»* (GF8-M-Enf-Assist-Hosp).

#### Subcategoría 3: Cambios Organizativos

Los cambios en el contexto organizativo también son importantes factores de riesgo psicosocial:

*Y hay muchos cambios todo el tiempo. La semana pasada es diferente de esta semana. Así que todo cambia muy deprisa. Así, que esto también acaba exigiendo una cuestión emocional muy grande* (GF6-M-Enf-Gestão-Cooper).

*Hemos tenido muchos problemas con los cambios, porque todo es nuevo. Todos los documentos se han modificado para satisfacer las necesidades actuales; desde la perspectiva de la empresa, para cumplir las políticas del hospital. Así que estamos bastante desbordados* (GF11-D-Enf-Gestão-Hosp).

*El hospital como un todo está pasando por cambios en este momento, pero también hemos tenido un cambio en el jefe de nuestro sector* (...) eso *crea inseguridad* (GF1-MP-Enf-Assist-Hosp).

#### Subcategoría 4: El Papel del Trabajador en la Organización

Existe la ambigüedad de funciones y responsabilidades en el trabajo, especialmente las obligaciones y presiones de ocuparse de la vida de otras personas:

*La presión en sí de que tengo que lidiar, tengo que portarme bien, porque es la vida de otras personas, ¿no? Siempre es el padre de alguien, el marido de alguien que está ahí en mis manos y tengo que cuidarlo* (GF8-M-Enf-Assist-Hosp).

*Hay situaciones que suceden que ni siquiera sé cómo resolver. Voy a tener que investigar para ver cómo puedo apoyar a este usuario* (GF12-D-Enf-Gestão-At-Primária).

*No ser pagado por lo que está haciendo, las posiciones que está ocupando allí. Y ser consciente de esta* (...) *esta devaluación financiera también* (GF6-DD-Enf-Gestão-Cooper).

#### Subcategoría 5: Desarrollo Professional

El desarrollo de carrera, como factor de riesgo psicosocial en el trabajo de estos profesionales que buscan cualificarse a través de estudios de posgrado stricto sensu, está relacionado con la falta de progresión y claridad en su carrera profesional, así como con la visión limitada del valor atribuido a su trabajo.

*Y no valoran la cualificación, así que creo que es un poco frustrante. Invertimos en nuestra carrera y no la valoran, aunque se aprovechen de nuestro talento, escritura, metodología* (...) *Me siento infravalorada* (GF4-D-Enf-Gestão-Secret-Saúde).

*A veces sólo te dan más trabajo porque estás más cualificado* (GF2-D-Enf-Assist-Hosp).

*Donde yo trabajo, no hay un plan de carrera* (...) *eso falta mucho, sobre todo la cuestión de incentivar a los que están dispuestos a estudiar para llevar adelante su trabajo y el incentivo financiero* (GF4-D-Enf-Assist-Hosp).

#### Subcategoría 6: Relaciones Interpersonales

Las relaciones interpersonales pueden ser difíciles en algunos contextos, ya sea con el personal, los directivos o los usuarios:

*Creo que no hay una comunicación adecuada* (...) *entre el equipo multiprofesional. Puede causar mucho malestar en la rutina de trabajo, acaba perjudicando al paciente y a veces genera estrés para el equipo* (GF2-M-Fisio-Assist-Hosp).

*E incluso el gerente, que es inmaduro, no tiene experiencia, no sabe cómo tratar con el personal, carece de empatía* (GF13-D-Cirur-Dent-At-Primária).

*El trato con el público es estresante. Cuando digo el público, me refiero a la gente, ¿no? Tanto los compañeros de trabajo como los usuarios en general* (GF12-D-Enf-Gestão-At-Primária).

#### Subcategoría 7: Interfaz Entre Trabajo y Família

La interfaz entre trabajo y familia se perfila por un viaje multifacético, que requiere la necesidad de equilibrar el trabajo, los estudios, la vida familiar/personal y también las responsabilidades domésticas:

*Es una triple jornada, contando vida doméstica, rutina de higiene, limpieza... trae un nivel de estrés muy alto y, a veces, un cierto agotamiento (GF13-DD-Psico-Clín).*

*No me siento muy satisfecha con el tiempo que puedo dedicar a mi familia, novio, amigos; me he distanciado mucho de mis amistades que antes tenía, simplemente porque a veces, trabajando seis días seguidos, con clases a los cinco días, no tengo energía social para ver a nadie* (GF11-M-Enf-Asistente-UPA).

*A veces siento esa insuficiencia de que me gustaría estar en más reuniones familiares* (...) *se siente como que no estoy 100% presente* (GF2-M-Fono-Clín).

#### Subcategoría 8: Acoso y Violência

Se ha demostrado que el acoso y la violencia son factores de riesgo en el lugar de trabajo en salud, al igual que la discriminación, el racismo y la intolerancia religiosa:

*Yo trabajo en una Unidad de Urgencias, y es una unidad rodeada de mucho barraco y falta de educación* (...) *lo que nos dificulta hacer nuestro trabajo* (GF11-M-Enf-Asistente-UPA).

*Tenemos violencia moral. A veces el usuario está descontento con el servicio y acaba ofendiendo al trabajador* (...) *Siento que estamos muy solos en términos de apoyo. La gente dice palabrotas, ofende, eso es todo, y tenemos que acostumbrarnos a ello, por desgracia.* (GF12-D-Enf-Gestão-At-Primária).

*En el hospital, he visto lo que es realmente el bullying* (...) *sobre todo por parte de la dirección de enfermería* (...) *la gente viene y utiliza un lenguaje soez, te dice palabrotas, te llama tonta, que no sabes. Y cuando vamos a hablar o a denunciarlo, tenemos miedo de que nos castiguen. Así que nos callamos, sufrimos y no queremos hablar para no arriesgarnos a que nos despidan, por ejemplo* (GF6-D-Enf-Assist-Hosp).

*Trabajo directamente con gerentes, directores, por lo que trato temas delicados del hospital. Incluso he tenido directores que me han pedido que cambie datos en el sistema, que cambie datos sobre pacientes hospitalizados, porque interferiría con sus ingresos. Cosas bastante pesadas* (GF3-MP-Enf-Gestão-Hosp/Docente).

*Sufrí intolerancia religiosa y racismo. Fue un momento muy duro también y me afectó durante todo el mes, sobre todo por el proceso de investigarlo, de tener que seguir reviviéndolo* (GF6-DD-Enf-Gestão-Cooper).

#### Categoría 2: Contenido del Trabajo

El contenido del trabajo se refiere a la naturaleza intrínseca de las actividades laborales y abarca los siguientes factores de riesgo psicosocial: entorno y equipos de trabajo; planificación de las tareas; carga y ritmo de trabajo; y esquema de trabajo. Estos factores se presentan a continuación.

#### Subcategoría 1: Entorno y Equipo de Trabajo

En lo que respecta al entorno de trabajo y al equipamiento, existen retos relacionados con la fiabilidad, la practicidad y/o los recursos para llevar a cabo el trabajo. Algunos posgraduados que trabajan se enfrentan a la falta de recursos materiales básicos, sistemas de información limitados e infraestructura en el área de la salud:

*Estamos pasando por un momento de mucha dificultad en relación a los recursos materiales, falta de material básico. Nos quedamos sin esparadrapo y sin micropore* [esparadrapo microporoso] (...) *sin 100 ml, 250 ml y 500 ml de suero fisiológico* (GF1-MP-Enf-Assist-Hosp).

*Hay que trabajar con lo que hay* (GF13-MP-Enf-Assist-Hosp).

*La velocidad de procesamiento del ordenador es muy mala. Internet no funciona, a veces se va la luz, ¿sabes? El baño más cercano está bastante lejos. No hay lavabo, por ejemplo, para lavarse las manos, ¿sabes? Es un órgano adaptado dentro de una estructura mayor* (GF12-MP-Enf-Gestão-Hosp).

#### Subcategoría 2: Planificación de Tareas

La planificación de tareas corresponde a la falta de oportunidades para adquirir nuevos conocimientos. Existe frustración debido a la falta de orientación y formación por parte de la coordinación y la dirección en determinados contextos laborales. Pero también hay desvalorización por parte del trabajador, que se muestra reacio a asistir a conferencias y sesiones de formación para adquirir conocimientos:

*Las cosas surgen y tenemos que hacerlas sin ninguna cualificación, sin ninguna formación para ello. Es muy estresante* (GF10-M-Enf-Assist-Hosp).

*En general, recibimos mucha gente vulnerable, gente viviendo en la calle, mucha demanda psiquiátrica. Así que hay muchos profesionales que no tienen el «tacto»* [formación] *con esta población vulnerable* (GF11-MP-Enf-Assist-UPA).

*Nos damos cuenta de que incluso la formación de este personal es difícil, porque nunca están disponibles para la formación* (GF11-D-Enf-Gestão-Hosp).

#### Subcategoría 3: Carga de Trabajo y Ritmo

Cabe destacar que la carga de trabajo y el ritmo también se destacan en salud, incluso en el trabajo autónomo:

*Incluso para el área de gestión, es un área muy exigente. Hay que estar muy presente; diferente, por ejemplo, de un coordinador médico. El coordinador médico, tiene el trabajo, pero no está «full time»* [todo el tiempo] *en el sector. Hoy en día, un coordinador de enfermería tiene que estar allí* (GF10-M-Enf-Gestão-Hosp).

*Lo que a veces dificulta es la cuestión del exceso de trabajo, que es prácticamente inherente a la profesión de enfermería, asumir muchas responsabilidades* (GF12-M-Enf-Assistão-Hosp).

*Así que hay mucho trabajo que me llevo a casa, estableciendo terapias, haciendo informes, estableciendo protocolos* (...) *no hay nadie que se ocupe de mi agenda* (...) *emitiendo notas. Así que también hago todas estas cosas burocráticas* (GF2-M-Fono-Clín).

#### Subcategoría 4: Horario de Trabajo

El horario de trabajo incluye horarios imprevisibles, largas jornadas laborales y turnos de noche. La falta de horarios fijos y la necesidad de cubrir diferentes turnos puede repercutir negativamente en la rutina y en el tiempo disponible para actividades fuera del trabajo, como en el caso de los estudios de postgrado. Esto añade complejidad e inestabilidad a la gestión del tiempo de estos profesionales:

*Los fines de semana, eventualmente nos llaman, como es la gestión* (GF1-D-Enf-Gestão-Secret-Saúde/Docente).

*Yo no tengo un horario fijo, soy un poco off-worker, entonces trabajo de mañana, de tarde, de noche, cuando necesito, y eso a veces dificulta mis actividades de posgrado* (GF2-D-Enf-Assist-Hosp).

*Trabajo por las noches. Así que a veces hay momentos en los que duermo todo el día... un sueño de cansancio extremo también, un sueño que no descansa* (GF11-M-Enf-Assist-UPA).

*Trabajo de noche, y durante el día estamos muy cansados* (GF13-MP-Enf-Assist-Hosp).

## DISCUSIÓN

De acuerdo con el análisis de los factores de riesgo psicosocial relacionados con el trabajo de los estudiantes de posgrado que trabajan en el área de la salud, este estudio encontró que los estudiantes de posgrado que trabajan enfrentan factores de riesgo psicosocial significativos en el contexto del trabajo en salud, que consecuentemente influyen en sus reacciones emocionales, con un impacto no sólo en el trabajo, sino que se extiende a otras áreas de la vida, incluyendo la académica.

Por lo tanto, es importante señalar que los factores de riesgo psicosocial pueden estar relacionados con el contexto laboral, en términos de cultura y función organizativa, así como con las prácticas de gestión. El comportamiento nocivo de un líder se asocia con el malestar psicológico de los empleados, lo que provoca efectos negativos que van desde la pérdida de autoestima, la reclusión, la inquietud, la evitación hasta la preocupación y la resistencia a aportar comentarios y contribuciones en el trabajo^(17)^. Un estudio cualitativo realizado en Noruega con maestros enfermeros reveló que las experiencias de los participantes son ambivalentes, ya que estos profesionales son amenazas al mismo tiempo que recursos en la organización del trabajo^(18)^.

Se ha observado que muchos profesionales de la salud, especialmente en el ámbito de los cuidados, se enfrentan a una falta de autonomía en el trabajo. Desde esta perspectiva, cuando existe un entorno de trabajo favorable en el que los empleados disfrutan de una autonomía adecuada, se sienten capacitados y motivados para alcanzar su potencial^(5)^, lo que contrasta con los resultados de este estudio, ya que algunos trabajadores se sienten desmotivados.

En cuanto a los recursos, este estudio mostró limitaciones, incluido el apoyo emocional a los trabajadores. Las prácticas de salud integrativa y complementaria*(*1) , la puesta en marcha de iniciativas centradas en la autogestión, el empoderamiento de los trabajadores y el acceso a servicios de salud mental, como sesiones de mindfulness o meditación, en el lugar de trabajo, pueden contribuir a reducir los niveles de estrés psicológico, ansiedad y burnout entre los profesionales^(19)^, por lo que son relevantes para esta población.

Sin embargo, la falta de disponibilidad de estos recursos, así como la sobrecarga de trabajo u otros factores de riesgo presentes en el contexto y el contenido del trabajo, pueden ser perjudiciales para la salud de los trabajadores. Además, las formas de abordar la dinámica y la estructura del trabajo sanitario suponen un reto y pueden provocar importantes enfermedades entre los profesionales. Este contexto se ve agravado por la complejidad del trabajo en salud, unida a las exigencias de gestión de las demandas académicas, que también presentan dificultades y exigencias^(9)^. Estas condiciones, tal y como están estructuradas, son fuentes potenciales de enfermedad.

Aunque la salud mental y la calidad de vida en el trabajo, especialmente entre los trabajadores sanitarios, se ven afectadas en su mayor parte por las estructuras organizativas y ocupacionales, que son gestionadas tanto por las organizaciones como por los propios trabajadores, se presta poca atención a las relaciones laborales capitalistas que perpetúan las condiciones insalubres del entorno profesional contemporáneo. En este contexto, es responsabilidad de las iniciativas de gestión y de los centros de formación abordar la salud mental en el trabajo, incluyendo cuestiones como la explotación, la alienación y la resistencia dentro del sistema capitalista^(20)^, incluidos los factores de riesgo psicosocial.

En cuanto a la toma de decisiones y el control, esta investigación ha demostrado que la falta de flexibilidad y de apoyo a las actividades académicas y al desarrollo profesional puede limitar la autonomía de los trabajadores a la hora de gestionar su tiempo y desarrollar sus capacidades. En este sentido, la gestión de las horas de trabajo, especialmente en un entorno hospitalario, es un reto debido al conflicto entre las necesidades de los empleadores y los deseos de estabilidad y previsibilidad de los trabajadores. Factores como el control percibido, el apoyo del supervisor, los cambios de turno y las horas extraordinarias afectan al agotamiento emocional y a las intenciones de rotación^(21)^.

Un estudio realizado en China sugiere que un mayor apoyo organizativo puede incluso mejorar la implicación en la investigación y reforzar la motivación intrínseca y extrínseca de los investigadores. De este modo, las universidades y los hospitales tienen el potencial de ofrecer educación sobre la importancia de la investigación en salud, lo que corrobora la motivación intrínseca y extrínseca para la investigación. En el caso de las enfermeras que cursan programas de máster, los hospitales pueden ajustar las políticas en función de sus necesidades, proporcionando apoyo organizativo para facilitar el proceso de aprendizaje de la investigación^(22)^. Sin embargo, en la práctica, existen barreras al apoyo organizativo que incluso pueden repercutir en la implicación de los posgraduados que trabajan en sus estudios y, en consecuencia, en su compromiso.

En lo que respecta a los cambios organizativos, se constató que, cuando se producen, pueden afectar psicosocialmente a los trabajadores. Los cambios mal gestionados o comunicados dentro de una organización pueden causar estrés debido a la incertidumbre y las dudas que generan en las personas^(5)^.

En relación con los roles organizativos, se han señalado ambigüedades en las funciones y responsabilidades, que constituyen importantes factores de riesgo psicosocial en el contexto laboral. Por lo tanto, es necesario garantizar que los trabajadores comprendan claramente sus funciones y las responsabilidades de los demás implicados en el trabajo^(5)^.

Por lo que respecta al desarrollo de la carrera profesional, en este estudio se observó una falta de reconocimiento y valoración de las carreras, especialmente de las titulaciones académicas. Un estudio realizado en línea con estudiantes de radiología y personal clínico y académico a escala mundial, en el que se obtuvieron 176 respuestas, reveló que la titulación de doctor no es necesaria para progresar en la carrera profesional para 126 (72%) de los encuestados^(23)^. Estos resultados son similares a los de este estudio, ya que para algunos participantes, la titulación no se tiene en cuenta para la progresión profesional, lo que genera sentimientos de desmotivación y falta de reconocimiento por parte de la organización del trabajo^(9)^.

En cuanto a la interfaz entre trabajo y familia, en las últimas décadas los países occidentales han experimentado importantes cambios demográficos, culturales y sociales que han afectado a la interfaz entre trabajo y familia. Estas transformaciones han puesto de manifiesto los retos que plantea conciliar simultáneamente las responsabilidades profesionales, familiares y domésticas^(24)^. Por lo tanto, se busca la conciliación de la vida laboral y personal para lograr una integración más armoniosa entre las esferas profesional y personal, pero es una barrera importante y, en este sentido, un factor de riesgo psicosocial.

Este estudio demostró que las relaciones interpersonales son otro factor de riesgo psicosocial derivado de las diversas relaciones, ya sea con los compañeros de trabajo o con los directivos. En el contexto de las organizaciones sanitarias, las relaciones interpersonales son esenciales. Los investigadores alemanes proponen que las medidas estructurales, como las reuniones periódicas de equipo y una formación adecuada, son útiles para mejorar la comunicación interprofesional de forma específica^(25)^. En esta línea de pensamiento, además del autocontrol y el equilibrio emocional de los implicados, es importante contar con líderes capaces de intervenir y negociar los conflictos en favor de la resolución y la armonía en el trabajo.

La presencia de bullying y violencia en el trabajo fue otro factor de riesgo psicosocial identificado en esta investigación, el primero derivado principalmente de diferencias de trato y atribuciones, así como de términos ofensivos, groserías y coacción para realizar prácticas no éticas, generando daño y sufrimiento moral, así como racismo e intolerancia. En este sentido, las profesiones sanitarias implican un intenso contacto diario con personas, especialmente pacientes, compañeros de trabajo y directivos. Estas interacciones también tienen el potencial de exponer a estos trabajadores a situaciones de *acoso* y violencia, factores de riesgo con graves consecuencias para los implicados^(26)^.

Respecto al contenido del trabajo, uno de los factores de riesgo psicosocial destacados fueron los ambientes y equipos de trabajo, que en este estudio correspondieron especialmente a la falta de insumos, infraestructura deficiente y sistemas de información desactualizados. Por lo tanto, mantener condiciones de trabajo adecuadas es fundamental para preservar el bienestar y el buen desempeño de los trabajadores, especialmente de los enfermeros^(27)^.

En relación a la planificación de las tareas, este estudio constató la falta de orientación y formación como iniciativas de gestión del lugar de trabajo. Sin embargo, desde una perspectiva diferente, se observó una falta de aprecio por la formación por parte de algunos trabajadores, según algunos de los gestores que participaron en esta investigación. Esto puede deberse a la reticencia o falta de voluntad de los trabajadores para asistir a conferencias y sesiones de formación que serían útiles para su práctica profesional^(28)^.

En lo que concierne a la elevada carga de trabajo y el ritmo de trabajo, algunos trabajadores pueden ocuparse de tareas muy exigentes, en las que se les plantean muchos retos y presiones. Sin embargo, estas exigencias pueden causar estrés si la persona siente que no puede hacerles frente o si no tiene suficiente control sobre ellas. A algunos trabajadores les resultará difícil admitir que tienen problemas debido a demasiadas exigencias, quizás tomándolo como un signo de debilidad o inadecuación^(5)^. Además, en el sector sanitario existe una carga de trabajo de enfermería que interfiere directamente en la calidad de los cuidados y en la calidad psicosocial de estos trabajadores.

En este estudio se constató que los trabajadores se enfrentan a horarios variables, cobertura de diferentes turnos y periodos de guardia, lo que se traduce en jornadas laborales impredecibles y prolongadas, incluido el trabajo nocturno. Las empresas suelen adoptar prácticas con salarios bajos y largas jornadas laborales para maximizar la productividad, lo que puede afectar negativamente a la salud de los trabajadores^(29)^. Además de interferir en la salud mental, esto también puede perjudicar la salud física, y la bibliografía muestra una asociación negativa entre el trabajo nocturno y la salud cardiovascular^(30)^.

Por lo tanto, es importante reconocer la naturaleza cambiante del trabajo en el siglo XXI y esforzarse por desarrollar intervenciones eficaces que ayuden a superar estos problemas^(29)^, haciendo hincapié en los riesgos psicosociales y los factores que los implican. Es necesario desarrollar propuestas de intervención y/o políticas públicas que promuevan ambientes de trabajo saludables, especialmente en el sector salud y en conjunto con las IES, sobre todo cuando los trabajadores simultanean jornadas laborales con estudios de posgrado.

Las limitaciones de este estudio incluyen *el* hecho de que fue realizado con trabajadores de la salud que también eran estudiantes de posgrado en una IES pública brasileña. Además, hubo predominio de enfermeros, escasez de otras categorías profesionales y recolección de datos en formato presencial y a distancia. Sin embargo, entre los puntos fuertes del estudio está la presencia de participantes que trabajan en instituciones con contextos diversos (atención primaria, secundaria y terciaria), lo que da credibilidad al alcance de los factores de riesgo psicosocial en salud.

Con relación a los avances de este estudio en los ámbitos de la sanidad y la enfermería, los gestores tienen la responsabilidad legal de garantizar que los riesgos se evalúen y controlen adecuadamente. Además, es pertinente la participación de los trabajadores en este proceso, para garantizar una identificación y gestión más eficaz de los problemas, además de ser un tema necesario para las intervenciones educativas y la incorporación en el plan de estudios de las IES en salud.

Se sugiere la realización de más investigaciones para futuros estudios, especialmente utilizando un abordaje cualitativo con este público de estudiantes trabajadores, incluyendo profesionales de diferentes categorías de salud, a fin de ampliar la comprensión de los factores de riesgo psicosocial en el trabajo. También se recomienda la realización de estudios longitudinales para analizar la evolución de estos factores a lo largo del tiempo, así como investigaciones que exploren estrategias de afrontamiento e intervenciones para promover la salud mental de los trabajadores.

## CONCLUSIÓN

El análisis de los factores de riesgo psicosocial relacionados con el trabajo de los estudiantes de posgrado que trabajan en el área de la salud refleja las particularidades de cada contexto y contenido de trabajo, organizaciones y niveles de atención, con énfasis en los factores presentados en el trabajo de enfermería, que también tienen el potencial de influir significativamente en el compromiso académico. Por lo tanto, es necesario que estos factores, además de ser manejados en el lugar de trabajo, sean incorporados y profundizados en los programas educativos de las instituciones formadoras. Esto significa integrar estas cuestiones en los planes de estudio de los cursos de pregrado y posgrado, con vistas a desarrollar/mejorar las competencias, sensibilizar y preparar a los futuros profesionales para hacer frente a los retos y peligros psicosociales que puedan surgir en su carrera, ya sea en la atención sanitaria primaria, secundaria o terciaria.

## DISPONIBILIDAD DE DATOS

Datos disponibles. El conjunto completo de datos que respalda los resultados de este estudio se publicó en el propio artículo.
